# Epigenetic Control of Gene Expression in the Normal and Malignant Human Prostate: A Rapid Response Which Promotes Therapeutic Resistance

**DOI:** 10.3390/ijms20102437

**Published:** 2019-05-17

**Authors:** Fiona M. Frame, Norman J. Maitland

**Affiliations:** The Cancer Research Unit, Department of Biology, University of York, Heslington, York YO10 5DD, UK; fiona.frame@york.ac.uk

**Keywords:** epigenetics, heterogeneity, prostate cancer, differentiation, tumor-initiating cells

## Abstract

A successful prostate cancer must be capable of changing its phenotype in response to a variety of microenvironmental influences, such as adaptation to treatment or successful proliferation at a particular metastatic site. New cell phenotypes emerge by selection from the large, genotypically heterogeneous pool of candidate cells present within any tumor mass, including a distinct stem cell-like population. In such a multicellular model of human prostate cancer, flexible responses are primarily governed not only by de novo mutations but appear to be dominated by a combination of epigenetic controls, whose application results in treatment resistance and tumor relapse. Detailed studies of these individual cell populations have resulted in an epigenetic model for epithelial cell differentiation, which is also instructive in explaining the reported high and inevitable relapse rates of human prostate cancers to a multitude of treatment types.

## 1. Introduction: Prostate Cancer Is a Heterogeneous Disease Governed by Episodic Genomic Rearrangements

Human prostate cancer has a deserved reputation for being amongst the most heterogeneous of human tumors [1]. Heterogeneity is reflected at the level of (i) gene expression, where not all the cells within a cancer mass express tumor markers to the same extent, (ii) at the genetic level, where multiple genomic rearrangements are present within the same mass of tumor and ultimately (iii) in patient response to common therapies, where tumors with an identical pathology show widely diverse responses to standard chemotherapy treatments. For example, fewer than 50% percent of cancers show a positive response to standard of care treatment such as docetaxel, despite all tumors containing replicating cells which microtubule poisons such as Docetaxel should be able to affect [2].

Whilst such heterogeneity is generally accepted, it is rarely reflected in molecular studies of the cancers, either at the phenotypic or genotypic levels [3,4,5]. The effects of heterogeneity have been previously discussed in some detail [1], but the many different cell types in a “tumor” include variable proportions of stromal (connective tissue) cells which we and others have shown to play a significant etiological role in the gene expression within the epithelial component of the cancer [6,7,8], but also minor cell populations, as a result of lymphocytic or macrophage infiltration, the proportion of which is variable within the tumors, and has been shown to influence/treatment responses and hence patient outcomes [9].

The current approach to tumor heterogeneity, deep sequencing of genomic DNA, has identified a degree of genomic and clonal variations, and increasing levels of mutations between treatment naïve tumors [10] and aggressive castration-resistant disease (CRPC) [11], which develops after the failure of androgen receptor inhibition therapies [12,13]. However, all of these studies are limited by the resolution and error rates of the sequencing procedures, to probably 90% i.e., a population of less than 10% within the tumor mass, is likely to be either overlooked or could fail to reach statistical significance [14]. A second historical limitation of these high resolution studies has been the need for relatively large amounts of DNA to carry out reproducible sequencing in the absence of G+C bias introduced by the need for PCR amplification at a sufficient depth of read to pick out rare populations, limiting the starting materials (such as those available in the online databases such as TCGA) to an analysis of larger tumors: imposing a selection for growth and often greater heterogeneity [15]. The ability to sequence single cell genomes should resolve this, but studies in other tumors [16,17] frequently turn up “novel” cell types. It is tempting to reject cells which do not fit an existing hypothesis, on the basis that they do not conform to an existing cancer phenotype [18], such as a luminal phenotype in prostate cancer.

In contrast to tumors where there is a known chemical etiological agent (e.g., components of cigarette smoke in bronchial carcinoma), prostate cancers have a relatively low rate of single base pair changes within the tumor mass [19]. In fact, most prostate cancers are characterized by episodic and catastrophic rearrangements of the genome, which include chromosomal translocations and segmental deletions [20]. The induction of such changes has been linked to androgen regulation via a number of different effector genes including the SPOP ubiquitin ligase [21,22].

**Figure 1:**
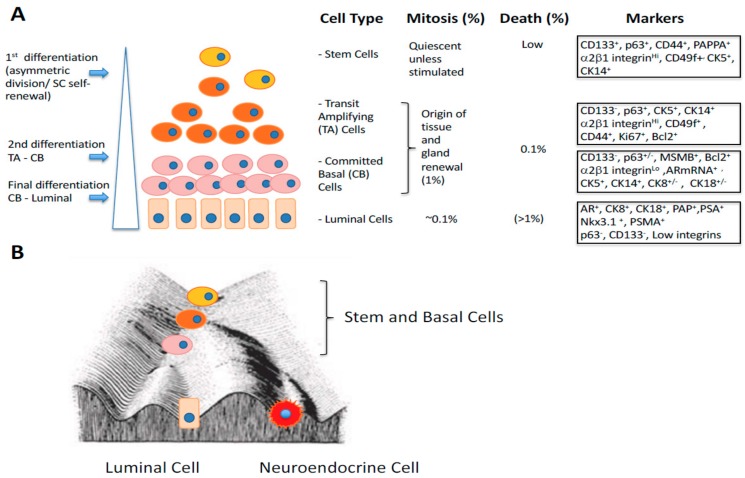
Cell phenotypes and the epigenetic landscape of normal human prostate epithelium (**A**) Epithelial Cell populations in a normal prostate, with the stem cell at the upper apex. It is important to note that the distinct cell types shown are simply representative and that the situation in a real tissue is much more plastic. Only the stem cell and the terminally differentiated luminal cell have truly defined phenotypes. The remaining cells are part of a continuum of change. Various antigenic markers used to enrich for or identify cell populations are shown in the extreme right column. (**B**) Waddington’s epigenetic landscape (after Waddington 1940). The model emphasizes the continuum of the differentiation process. The ultimate fate of a cell is to migrate to the base of the slope, as fully differentiated luminal and neuroendocrine cells. The reversibility of the differentiation continuum, to a stem cell fate from fully differentiated cells, requires “energy” or multiple gene mutations/activations to promote a cell “upwards” to the stem cell (SC) state.

Gene expression patterns are equally heterogeneous in both normal and malignant prostate glands where, as a cell becomes more defined, gene expression is not only more restricted, but is considerably enhanced. For example, the expression of genes for proteins secreted by the non-dividing, terminally differentiated prostatic luminal cells (e.g., polyamines and kallikreins) is governed by promoters whose activity is many thousand-fold higher than those of maintenance genes such as those required in basal cells (Figure 1). Some malignancy signatures more closely related to “immunity genes” may reflect lymphocytic infiltration levels whilst measurements of presumably tumor-derived RNAs in serum display virtually no identity in the diagnostic gene panels between duplicate studies of the same patient blood samples in different laboratories [23,24].

**Figure 2:**
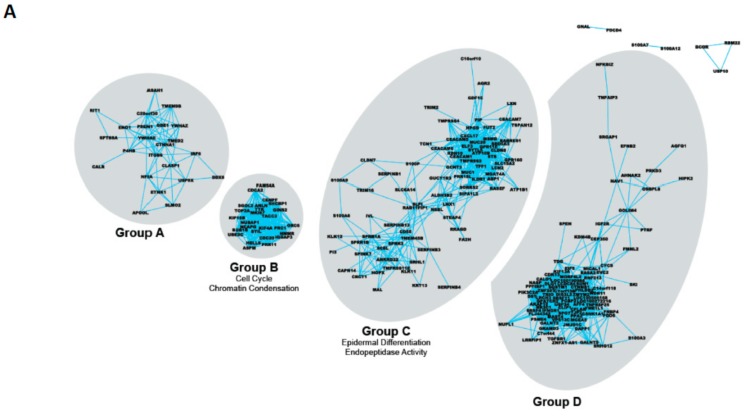

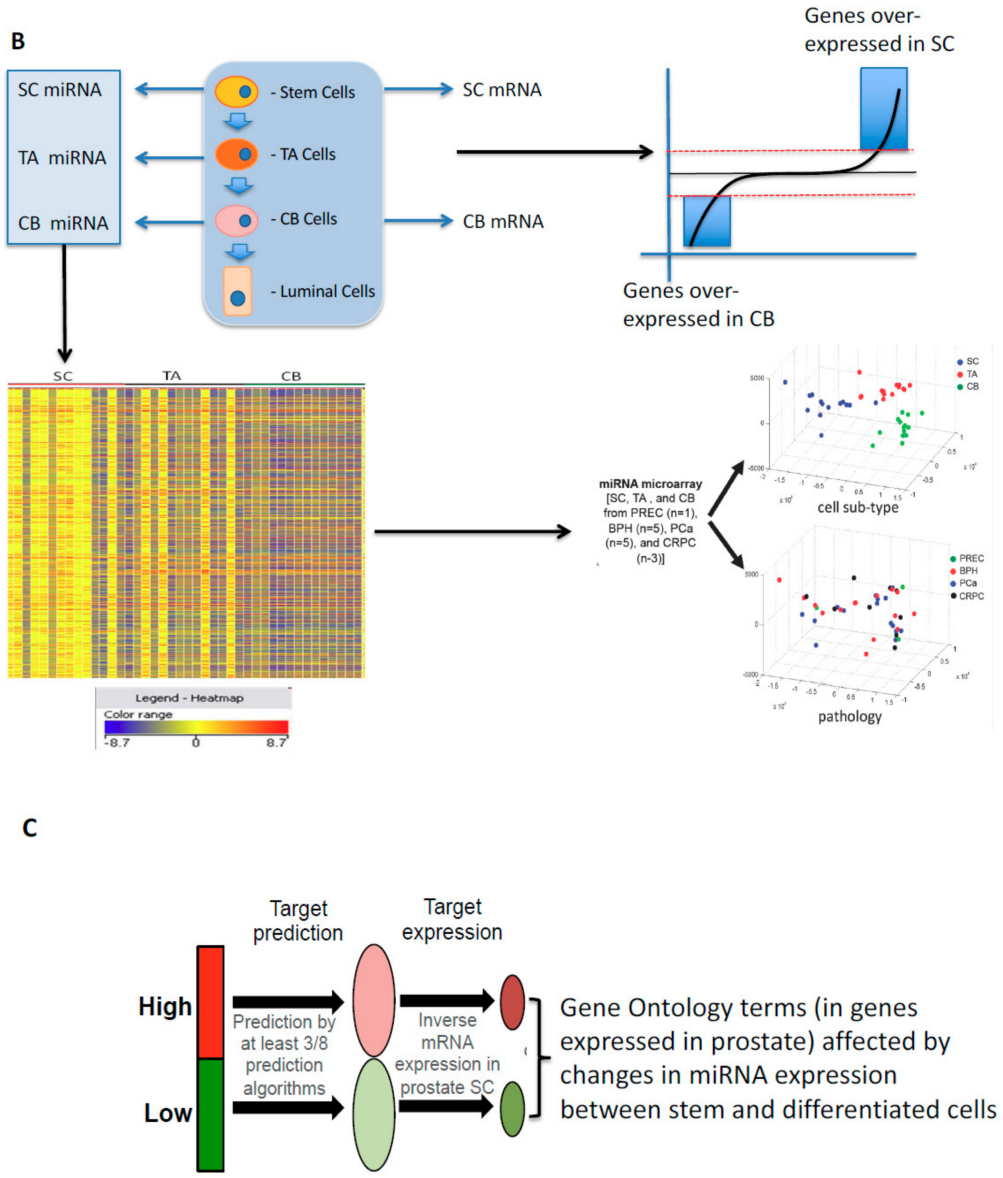
Strategies to categorize the genes and miRNAs which modulate prostate epithelial differentiation. (**A**) mRNA co-expression analysis in the transition from SC to CB (committed basal) cells in human prostate epithelial cells [26]. Clustering reveals gene pairs which always change together, in this transition and in other cell systems. Note the lack of overlap between the various gene clusters, implying a common control system for each cluster. Selected individual genes are listed in Table 1 and Table 2. Identification of individual genes is possible by zooming in electronically on the figure. (**B**) miRNA expression datasets were generated from different cell populations from prostate tissues with a variety of pathologies [27]. Unsupervised clustering of the miRNA database indicated that miRNA expression was more closely correlated to differentiation that normal/benign/cancer tissues. Note (lower right panel) the high expression of miRNA (warm color) in the SC, which decays in the TA population, with increasing downregulation (shown as blue) as the cells become more differentiated (CB cells) and begin to express a wider and more specialized mRNA set. (**C**) Gene ontology strategy to relate miRNA expression to that of mRNA known to be expressed in the various prostate epithelial cell types [28].

Even in human tissues where great care has been taken to micro-dissect the epithelial component, there remains a gross element of heterogeneity according to the basal or luminal origin of the epithelial cells. In normal prostate, the luminal epithelial fraction comprises around 50% of the cellular epithelial population, whereas basal epithelial cells form the remainder within a gland. In cancers, this ratio is extremely skewed: more than 99% of the cancer mass is a luminal-like cell, which is incompletely differentiated (whilst expressing androgen receptor and prostate-specific antigen) but has gained the ability to replicate in an uncontrolled fashion. However, within the tumor mass, there also remains a small population (fewer than 1% of cells) with a basal-like phenotype [1]. To resolve such minor populations requires an approach pioneered by John Dick and colleagues for live cell fractionation of Acute Myeloid Leukemias [25]. Given the complexities of cell phenotypes (see below) such approaches are at best high enrichments rather than purifications, but by analyzing the transcriptomes of the different populations the true phenotypes of minor populations can be derived and linked to function (see Figure 2).

Others have reported similar cell fractionation protocols, which indicated that a basal cell was the likely origin for the most aggressive and malignant prostate cancers. A human basal cell of origin [29,30,31] is more likely (but as yet unproven) than the luminal precursor predicted from mouse experiments [32]. To generate the predominantly luminal phenotype of prostate cancers, such a basal origin implies that the initiating cell population must both differentiate and expand. Until recently, the factors which control both normal and aberrant differentiation in prostate epithelium were unknown.

## 2. Stem Cell Versus Stochastic Mechanisms of Cancer Induction

The cell of origin (COO) of prostate cancer has been briefly discussed above. Logically, given the time required to develop a cancer, the COO should be long-lived in order to accumulate necessary changes. The slow turnover of prostate stem cells can also explain the slow progression and late-onset of prostate cancer (reviewed in [1]). Mathematical modelling by Tomasetti and Vogelstein [33] more recently sought to establish a positive correlation between the number of stem cell divisions and the incidence/age of onset of cancer in more rapidly renewing tissues such as the colon. Obligatory carcinogenic changes in pre-cancer [34] should include the loss of normal microenvironmental control (aberrant growth), although activation of the immune recognition of such emerging cell clones would be designed to delete such cells. The intracellular surveillance system (p53-mediated) for unscheduled DNA replication is also a necessary target for inhibition [35], as is the nature of the cell telomeres, whose erosion leads to senescent deletion of cells with critically shortened telomeres [36].

The cancer stem cell (CSC) hypothesis states that not all of the cells in the tumor are able to reconstitute the tumor mass after dissociation and that a sub-population of cells are responsible for tumor initiation, and post-treatment relapse. The CSCs have been termed stem-like cells, since their properties, and some phenotypic characteristics replicate the tissue-renewing stem cells in normal cell systems. It is assumed that the pool of CSCs, which is between 0.01% and 0.1% of a prostate cancer mass lies at the apex of an aberrant differentiation hierarchy (see Figure 1).

The more traditional view of carcinogenesis is embodied in the stochastic mechanism which proposes that the definitive carcinogenic event is initiated by an oncogene or tumor suppressor gene. The mutation is “fixed” in the population by subsequent mitosis, resulting in a daughter cell whose tumor propagation potential matches that of the initiating cell. Subsequent mutations and accumulation of different genetic lesions within the initiated “field” produced clones with growth advantages that can be traced to the founder event. There is now increasing evidence in prostate cancer (reviewed in Reference [37]) to show that epigenetic inactivation (CpG methylation ratios) of APC and RARβ genes in malignant tissues and adjacent histologically normal cells, imply the existence of an “epigenetically activated field” of cells [38], as seen in oral squamous cell carcinomas [39].

It is likely that the CSC and stochastic hypotheses are not mutually exclusive. Experimental evidence requires the initiating events to occur in longer lived and largely quiescent stem/progenitor cells, establishing pre-tumor development [34] which may be histologically undetectable, and provide a longer-term pool of “reserve cells”. However, in later-stage or aggressive disease a CSC-derived dominant clone, which shares founder mutations with the original clone but has enhanced proliferative and invasive capabilities could drive the cancer. Genetic heterogeneity and phenotypic plasticity can also be maintained in the original CSC pool to allow new and evolutionarily successful clones to develop in response to changes in microenvironment, such as treatments [40].

## 3. Gene Expression Changes During Epithelial Differentiation in Normal and Malignant Prostate

Since our original hypothesis was that in both normal and malignant prostate, the bulk of the cells is derived from an original (basal) stem cell [29], it is important to understand the controls which affect stem cell differentiation within the basal compartment. Whilst many markers have been devised to distinguish between the basal and luminal compartments (mostly upregulated genes in the luminal compartment), relatively little is known about gene sets and their changes in expression as a basal cell matures from an initial stem cell. To approach this, we exploited a gene expression data set [41], which defined gene expression within the putative stem cell population (CD133+/α2β1integrin high/CD44+) and the more differentiated basal cells loosely grouped into a committed basal cell type (defined as CD133-/α2β1 integrin low/CD44 high) (Figure 1A). We adopted this approach to minimize overlap within the continuum of change which is normally seen between stem cells and those basal cells committed to differentiation as shown in the figure, and to overcome the limitations of cell surface phenotype base enrichment where a “pure” stem cell population is likely to contain about 5% TA cells [42]. Luminal cells are also likely to contaminate the basal cell population, although by adopting a short-term adhesion and growth in culture strategy, luminal cells do not normally survive [43].

Our second assumption was that genes whose expression is changing during the differentiation process ought to be co-regulated in some way. Therefore, we re-analyzed our initial gene expression data on the basis of a high pair-wise correlation (Figure 2A) i.e., genes whose expression always moved up or down with at least one other gene in the group. We were able to identify four interaction networks of genes, which clustered closely together, Group A, B, C and D genes [26] (Figure 1A, Table 1). There were two additional striking features of this analysis. The first was that the four groups of interactomes were quite distinct, with no interconnections as often found in similar studies. Secondly, all four groups corresponded quite closely to particular elements within The Gene Ontology with the exception of Group A—the least tightly associated gene cluster [26]. Group B genes were associated with cell cycle and chromatin condensation, Group C, epidermal differentiation and endopeptidase activity, while Group D contained genes previously associated with cellular development. To extend the correlation, we analyzed a compendium set of 24,536 human Affymetrix gene expression microarrays from 800 experiments available online, containing expression data from at least 150 different human cell types, i.e., extending the gene co-expression to beyond just prostate epithelial differentiation. The final dataset contained about 1.2 billion expression correlations and clearly indicated that our initial grouping of co-regulated genes, based solely on prostate epithelial differentiation, was retained across many human tissues, implying that the control factors for such genes are highly conserved within different human pathologies and human differentiated cell types. Multiple transcription factors and a variety of steroid hormones were readily identified as potential primary regulators of the differentiation-associated gene sets in the prostate, for example, the tightly clustered Group C genes (associated with epidermal differentiation) [44,45]. Androgens, which control the terminal elements of prostate differentiation [46] had no discernable effect within the basal compartment whereas they were the primary driving force behind gene expression in purely luminal cells such as the LNCaP cancer cell line. The high biological activity of RA is perhaps not surprising, given its well-known effects on (i) cellular differentiation [44,47] (ii) cancer treatment (reviewed in [48]), and (iii) its potency in re-aggregation cultures of prostate cancers [49]. It was concluded that both retinoids and androgens are required to promote functional differentiation and that there should be a number of genes which are dually regulated by retinoic acid and androgens as the prostate differentiation pathway process proceeds [50]. Within the promoters of these genes e.g., the prostate transglutaminase (TGM4), the AR binding sites are in sufficiently close apposition to the pre-occupied retinoic acid binding site to imply that as androgen receptor levels in the nucleus increase, the bound retinoic acid response complexes are deposed by the AR complexes, resulting in a proposed switch for retinoic acid control to androgen receptor control [50].

However, such simplistic mechanisms cannot explain all of the co-regulations seen within the four expression groups during basal cell differentiation. An equally powerful regulator of co-expression would also seem to be high levels of a restricted number of transcription factors (Table 2) which could act either in combination or individually to regulate different genes. For example, ROCK2 has also been previously implicated in prostate cancer differentiation [51], and indeed inhibitors of Rock are used in specialist media to preserve a stem cell-like phenotype [52]. The range of these “master” transcription factors which are overexpressed during the differentiation process is listed in Table 2.

In terms of gene expression responses and co-regulation, the TMPRSS2 gene (frequently regarded an androgen “specific” gene, found in an active fusion with the ERG oncogene in about 50% of prostate cancers) [53] was clearly upregulated by androgens (12 fold), as expected, in the luminal compartment, but showed an even higher upregulation (40 fold) within the basal compartment from a base state under the influence of retinoic acid [26,54]. This is entirely logical, given the transcription profile of TMPRSS2, which clusters with HTGP4 and since TMPRSS2 is expressed at detectable levels in other non-prostate tissues, which do not express AR [50], according to the Human Protein Atlas: https://www.proteinatlas.org/ENSG00000184012-TMPRSS2.

Identification of the cognate binding sites within promoters for transcription factors and the range of differentiation-associated transcription factors (Table 2), has posed the question: What is controlling the expression of the differentiation regulatory transcription factors? Based on epigenetic controls known to act on such differentiation cascades in embryonic tissues, these are unlikely to be mutagenic effects or indeed mediated by genome re-arrangement, since they occur both in normal prostate and in the malignant equivalent. The control requires flexibility, reversibility and immediacy of action in cells to changes in micro-environment, ultimately resulting in prostate epithelial cell differentiation.

## 4. Epigenetics: A Mechanism for Phenotypic Flexibility in Development and Disease

Most current therapies for prostate cancer are directed against replicating differentiated cells [55], but the unique properties of a stem-like population render the latter undifferentiated cells resistant to virtually all treatments (see below). However the ability to differentiate appears to be hard-wired into all such stem-like cells, therefore one solution would be to deplete or eliminate the stem cells by inducing differentiation which is aberrant in prostate cancers [48]. This strategy works clinically in leukemia, where pre-treatment with retinoic acid results in a more differentiated cell, which can then be killed by traditional chemotherapies [56]. Similarly, the use of epigenetic manipulators of undifferentiated state such as histone deacetylase inhibitors [57] has been exploited to deplete the stem-like population in experimental breast cancer [58].

## 5. Defining Epigenetics in Human Genetics: The Elegance and Simplicity of Waddington’s Concept

In a purely theoretical analysis of cell fate determination in development, Conrad Waddington [59] visualized his “epigenetic landscape” as an inclined surface with lower branching ridges and valleys (Figure 1B). Waddington’s model provided a connection between genotype and phenotype, which in his opinion was not rooted in mutation (during development) but rather defined epigenetics as “Heritable changes in gene expression (active vs inactive genes) which does not involve changes to the underlying DNA base sequence i.e., a change in phenotype without a change in genotype’. The reversibility of the differentiation continuum or rather its effects on phenotypic plasticity has been elegantly demonstrated in induced pluripotent stem cells [60] which requires “energy” or multiple gene mutations/activations to promote a cell “upwards” to the SC state.

Waddington formulated these hypotheses in the complete absence of any potential epigenetic mechanisms in development or indeed cancer [61]. We now possess an appreciation of the diversity of such control via three mechanisms (i) DNA methylation, principally, but not exclusively at CpG sites, often in clusters within the human genome (ii) the status of chromatin (condensed, relaxed) which is determined by the modification status of the histones (acetylation, methylation) bound at specific sites and (iii) the expression of non-coding RNA such as microRNAs (miRNA) which can control the expression of multiple genes by recognition of target sites in mature mRNA, resulting in their destruction by potent and specific ribonuclease complexes. The relevance of epigenetic control in cancer development has recently been reviewed [62] and epigenetic changes, in the absence of common mutations, seem to underpin the development of malignant pancreatic cancers [63].

## 6. Epigenetics as a Flexible Response to Environmental and Microenvironmental Changes

In evolutionary terms, a permanent change such as mutation or deletion of a gene is clearly a strategy for many species. In cancer, however, a permanent response to drug treatments such as AR gene amplification/mutation [64] or DHFR amplification in response to methotrexate [65] cannot be undone when the treatment is withdrawn, or a compensation pathway is activated in the cancer cells. Normal and cancer stem cells are designed to survive multiple environmental challenges over a lifetime, and epigenetics provides rapid and flexible responses that are required [66].

## 7. The Epigenetic Landscape in Prostate Cancer

Returning to Waddington’s original Landscape, on to which the various epithelial cell types in normal and malignant prostate have been superimposed (Figure 1B), it is clear that substantial “energy” is required to promote a fully differentiated luminal cell into a stem-like cell [61]. Current attempts to achieve this by iPSC techniques have not been successful [67], and in the laboratory, whilst there is some plasticity between TA and SC, and a lower degree from CB and SC, luminal cells generated in vitro appear programmed to secrete “prostate-specific” products and senesce (Figure 1A) [68]. However, whilst this holds true for normal epithelial hierarchies, there may be a higher degree of plasticity in a tumor cell: where nevertheless biological energy must be spent to achieve the transition to a stem-like status.

## 8. Small Non-Coding RNAs: The Rapid Reaction Force for Environmental Changes in Differentiation and Cancer Treatment Suppressor miRNAs and Onco-miRNAs: Designed or Selected for Cancers?

Small non-coding RNAs, such as the more than 2000 microRNAs (miRNA) encoded in the human genome are a major mediator of epigenetic gene expression control [69]. Bearing in mind the earlier data indicating that cell fate decisions in prostate basal cell differentiation requires simultaneous control of a number of genes, and in particular a defined suite of transcription factors, specific miRNAs are good candidates to provide this level of simultaneous gene regulation. Specific miRNAs affect cell fate (and gene expression) in both human embryonic stem cells and induced pluripotent stem cells (reviewed by Reference [70]), and miRNA levels are generally higher in the least differentiated (stem cell) populations.

Increased miRNA expression has also been linked to oncogenic changes—suppressing tumor suppressor genes there (onco-miRNAs) whereas some are downregulated, permitting the expression of known oncogenes (suppressor miRNAs). Unlike many other studies in common tumor types, there has been little consensus about the most significant miRNA expression changes in prostate cancer (reviewed by Reference [71]), whilst individual miRNAs have been associated with poor outcomes in prostate cancer patients [27] and cell lines [72,73].

When miRNA levels were studied in purified cell fractions from multiple normal and malignant prostate tissues (Figure 2B), there was no relationship between miRNA expression patterns and pathology, whereas unsupervised clustering indicated a close relationship to epithelial differentiation (the most significantly altered miRNA species are shown in Table 3). As frequently reported in ESCs, the prostate stem cells (of both malignant and non-malignant origins) expressed the highest levels of miRNA, which decreased as the cells differentiated and more gene sets were transcribed in the differentiated luminal cells [28].

## 9. Developmental Changes in miRNAs in Prostate Epithelial Cells of Normal and Malignant Origins

The miRNA expression profiles showed that (i) a miRNA signature was conserved in stem cells from BPH, PCa, and CRPC, implying that miRNAs play their most important role in regulation of essential SC properties such as self-renewal, prolonged proliferation, and a hard-wired capacity to differentiate, regardless of the pathological origins of the cells. (ii) The prostate SC miRNA signature overlapped considerably with that from human embryonic SCs (hESCs) [74] e.g., high expression of the miR-302 and miR372 families but suppression of the let-7 family (Table 3). (iii) The miRNA expression profiles of SCs and those of previously published unfractionated CRPCs [75] overlapped by approximately 60%. The shared suppressed miRNAs have previously been shown to regulate key SC and cancer-associated proteins such as c-MYC, KLF4, NANOG, and EZH2 in SCs and CRPCs. (iv) The PCa-cancer stem-like cell (CSC), CRPC-CSC, and normal SC populations each had significantly different miRNA expression signatures, which distinguished them from their more differentiated cell progenies.

Given the strong link between differentiated state and miRNA expression in this study, a number of the previously prostate cancer (both primary and CRPC) -associated miRNA identified in Rane et al., [27] had previously been reported to be onco-miRs and tumor suppressor miRNAs, such as (i) miR-629 and miR-203 [76,77] (ii) suppression of miR-299-5p, which is down-regulated [78] in metastatic cell lines compared with normal prostate epithelial cells, and (iii) the CSC signature identified miR708 whose suppression in prostate xenograft cells unregulated both CD44 and Akt [79], whereas the CRPC signature implied that miR-521 was implicated in a radio-resistant LNCaP phenotype) [80].

Finally, since multiple miRNAs implicated in the hESC maintenance program are conserved in adult human prostate epithelial SCs, this strengthens the evidence for a stem-like status for CD133+/CD44+/a2b1 integrin high cells—a status which is ultimately acquired by malignant cells in CRPC after multiple rounds of androgen-based therapies, emphasizing the basal stem-like nature of these advanced. and broadly incurable cancers [81].

Bioinformatic analysis to identify the most significant changes in expression has recently been compromised in unfractionated tumor biopsies in a paper describing the expression of the onco-miRNAs 143/145 in normal mouse colon [82]. Previously considered to be markets of malignant epithelium, a cell-specific study found that the major 143/145 expressing cell type was a stromal cell, although this did not seem to be the case in prostate epithelial cells [27,83]. Changes in miRNA expression using RNA from heterogeneous cancer biopsies were, therefore, a result of changes in the stromal content of the biopsies.

We sought to minimize the impact of our study of epithelial differentiation, by restricting the candidate genes suppressed by miRNA control after referral to a range of existing algorithms (MiRWALK) to those which were actually expressed in prostate epithelium [28]. Secondly, rather than focus on individual genes, we correlated the changes in miRNA expression and their target genes to cellular functions, exploiting the Gene Ontology Database. Again, we used co-expression patterns to cluster the miRNAs, and identified seven groups, which were related in their mRNA targets, and the seed sequence similarities [84]. Since miRNAs of known similar functions (e.g., let-7 and miR-17-92 families) did cluster together, we identified significant functions with increased confidence. Both Notch signaling and DNA damage repair emerged as the outstanding significant functional candidates (Figure 2C) for epigenetic control by miRNA during prostate epithelial differentiation [28].

## 10. Phenotypic Plasticity and a Stem-Like State as a Mechanism for Radio-Resistance

It has been suggested in studies of other cancer types, that both the induction of a more stem-like state and chromatin status play a dual role in radio-resistance [85,86,87,88,89,90], as we have shown in primary cultures of prostate epithelium (see below). The separate and unbiased discovery of DNA damage repair as a major miRNA-controlled function in prostate differentiation [28] prompted a search for potential candidate genes whose expression might be specifically regulated.

Phenotypic plasticity, and the generation of cells with a stem-like phenotype from more differentiated cells, has also been implicated in therapy resistance. Since our miRNA dataset implied such a close relationship between treatment-resistant cells (CRPC) and that miRNA could perturb differentiated state. Here, our candidate selection was driven by the set of transcription factors identified earlier: i.e., RXR, Rock2, vitamin D receptor, GCRs, TAZ serum response factor and HSF1. Within the set of miRNA over-expressed in SC, one miRNA: miR548c-3p had a high potential to control the differentiation-linked transcription factor expression set. This miRNA is overexpressed approximately fivefold in SCs compared with more differentiated cells, and its overexpression has been associated with poor patient survival [76]. When miR548c-3p was overexpressed in CB cells (i) a more stem-like phenotype (CD49b (integrin b2) and CD49f (integrin b6)) was generated (ii) colony-forming efficiency (an indicator for SC self-renewal capacity) increased by 75% (iii) mRNA expression of multiple SC-regulated genes (e.g., up-regulation of NFKB ID2 PROM1 and SOX2) with a simultaneous reduction in CB cell-specific genes (CEACAM6, WNT5A, LCN2), but perhaps most significantly (iv) the radiosensitive CB cells became more resistant to 5Gy of x- irradiation [27]. Over-expression of miR-548c-3p has previously been shown to reduce doxorubicin-induced DNA damage in HeLa cells by inhibition of DNA topoisomerase IIa [91]. This is a clinically relevant result since in untreated tissue-derived CRPC cells, miR-548c-3p was significantly unregulated compared to benign cell-derived epithelial cells and higher serum levels of miR548c-3p were found in CRPC patients compared to serum from low-risk PCa patients [92].

## 11. Increased Heterochromatin and Rapid Chromatin Condensation as a Mechanism for Radio-Resistance in Prostate Cancer Stem Cells—The Role of Histone Modifications

Stem cells contain bivalent or “poised” chromatin, which share active and repressive histone binding [93,94]. The distribution of such marks changes radically as the stem cells differentiate and differentiation-specific genes are activated. In embryonic stem cells, which have a high degree of pluripotency, the existence of bivalent chromatin marks permits rapid activation or repression of genes required during the first essential step in differentiation i.e., self-renewal [95,96].

**Figure 3:**
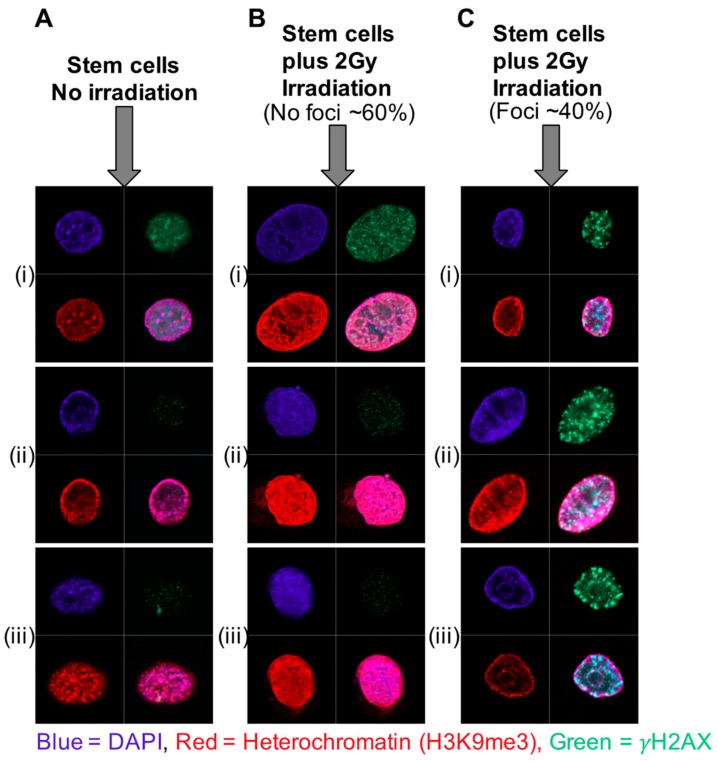
Nuclear architecture and foci of DNA repair in prostate epithelial cell subtypes. Primary prostate epithelial cells were cultured and separated into stem cells (SCs), transit amplifying cells (TAs) and committed basal cells (CBs). Each cell population was irradiated with 2Gy irradiation and cells were fixed and stained 30 min post-irradiation for nuclear stain (DAPI) (blue) heterochromatin (H3k9me3) and γH2AX (green). Unlike TA and CB cells, where the majority of cells (~80%–95%) showed DNA damage (γH2AX foci) post-irradiation, only ~40% of SC were positive for foci. This trend was observed in 13 BPH (benign prostatic hyperplasia) samples and 15 prostate cancer samples. The SCs that were negative for foci had a distinctive heterochromatin pattern. In the SCs that were positive for foci, the foci did not coincide with the regions of heterochromatin. Images represent (**A**) unirradiated stem cells with no foci, (**B**) irradiated stem cells with no foci (60% of cells) and (**C**) irradiated stem cells with foci (40% of cells). Three examples of each group are shown (i), (ii) and (iii) [100].

The existence of condensed or bivalent chromatin has been proposed as a response and resistance mechanism to a number of therapies for cancer [97,98,99]. Primary epithelial cultures of stem-like cells derived from patient prostate tissue (CD133+/α_2_β_1_integrin^hi^) sustained less DNA damage and were, therefore, more resistant to radiation than the more differentiated transit amplifying (TA) and committed basal (CB) cells [100]. In retinal rod cells [101], preferential localization of radiation-induced DNA damage foci was seen in euchromatin whereas the nuclei of untreated stem cells in primary prostate epithelial cell cultures presented with higher levels of heterochromatin (and less euchromatin) than the TA and CB cells, as shown by staining for histone marks H3K9me3 and H3K27me3, using immunofluorescence and flow cytometry. Following low dose irradiation (2Gy), the heterochromatin rapidly adopted a distinctive and highly condensed conformation, which was not observed in the more radiation-sensitive TA and CB cells. There was a lower overall percentage of cells positive for DNA damage foci post-irradiation, but the cells that did stain for DNA damage foci showed no overlap of heterochromatin and foci; i.e., any foci were located in areas of euchromatin (Figure 3). We reasoned that chromatin de-condensation should increase DNA damage and therefore the effectiveness of radiotherapy for the stem cell fraction. Pre-treatment of the SC population with a low (non-cytotoxic) dose of Trichostatin A (TSA), a histone deacetylase (HDAC) inhibitor, increased the radio-sensitivity of the SC fraction by 20%–40%, whilst not affecting the more differentiated and radio-sensitive cells. A significant synergy with radiation after treatment with another (clinically approved) HDAC inhibitor, Vorinostat (suberanilohydroxamic acid—SAHA) was observed in multiple primary prostate epithelial cultures [102] as defined by colony forming ability.

Like many other stresses, hypoxia [103] also results in alterations to transcriptional regulation resulting from epigenetic changes including chromatin remodeling [104]. Hypoxic conditions have been shown to induce chromatin compaction in some cells, those undergoing arrest and apoptosis, and induction of bivalent chromatin [105]. If this occurs within the tumor, it could also contribute to the radio-resistance mechanisms within the hypoxic region. Overall, the current data for prostate cancer strongly promotes the use of the HDAC inhibitors (at sub-cytotoxic doses) as radiosensitizers in a combination treatment in much the same way as exploited in clinical trials for other cancer types [106].

## 12. miRNA-Induced Changes in Chromatin Status as a Mechanism for Radio-Resistance in Prostate Cancer

Many miRNAs play a variety of roles in resistance to chemotherapy, and the Gene Ontology link from our linked profiling of mRNA/miRNA implied that the same feature would apply to radiotherapy-induced DNA damage repair [27,28]. miR-99a/miR-100 was one candidate family downregulated not only in SC and CSC but also in patients with CRPC (compared to benign disease). Furthermore, patients with low levels of miR-99a/miR-100 are more susceptible to biochemical recurrence after treatment [76].

When expression was studied in both cell lines and primary tissue-derived cells, inhibition of miR-99a/miR-100 prevented p53 dependent apoptosis in irradiated PCa cells. In a similar manner to HDAC inhibitor treatment (see above), suppression of miR-99a/100 results in relaxation of damaged chromatin by both histone acetylation and recruitment of DNRCA1 and RAD51 [107]. Of the mRNA targets of miRNA99a/100, SMARCA5 and SMARD1 were identified at the principal drivers of resistance [107,108]. No effects were seen on stem cell phenotype, despite previous studies showing that the related SMARCA3 cooperated with the relevant transcription factor EZH2 in epithelial stem cell maintenance [109].

Expression of PARP1 whose inhibition was recently shown to increase survival times a subset of PCa patients [110], is another essential component of the miR-99a/100 driven DNA damage response [111]. When PARP inhibition was tested as a potential enhancer of radiotherapy in PCa using either siRNA against PARP1 or nicotinamide (a non-selective PARP inhibitor), recruitment of both SMARCA5 and, to a lesser extent, SMARCD1 was inhibited [108]. Thus, the established PARP activity forms a radiation resistance network with the miR-99a/100-SMARCA5/SMARCD1 axis. The more commonly observed induction of epithelial-mesenchymal transition [112] as a resistance to PARP inhibition or miR99a/100 manipulation was not observed in our studies [108]. We also showed that expression of miR99a/100, like miR708 in ovarian cancers [113] was susceptible to control by hormones, most notably glucocorticoids, by an as yet undetermined but probably indirect transcriptional control mechanism. This raises a serious clinical question about radiotherapy (and chemotherapy) treatment protocols, where patients are often administered glucocorticoids to counteract the side effects of radiation. Clinical studies have demonstrated that GCR inhibition promoted docetaxel resistance in PCa [114], and GCR is expressed at significant levels in the chemo- and radiotherapy resistant CSCs [41], after pre-treatment with Dexamethasone or the GCR inhibitor. In our experiments on near patient samples, the GCR inhibitor mifepristone produced sensitivity changes of primary PCa to irradiation, by directly influencing the expression levels of miR-99a/100 [108]. Therefore, the use of GCR inhibitors rather than glucocorticoid supplements should clinically enhance radiotherapy and perhaps reduce tumor relapses.

## 13. The Paradoxical Role of Genomic Methylation in Prostate Epithelial Differentiation and Carcinogenesis

Control of cell fate really cannot be explained by any single transcription factor (see Table 2). Whilst activation of gene expression by suppression of miRNAs (see above) has the potential to affect many genes simultaneously, it is likely that binding of modified histones to transcriptional control mechanisms provide a form of reversible primary control in gene regulation, for example as demonstrated in radiation resistance.

Persistent changes to DNA are stabilized by CpG hypermethylation in many cancer types (reviewed by Reference [62]) including primary prostate cancers [115], although demethylase activity offers a degree of flexibility [97]. In embryonic and other stem cells, hypomethylation with foci of hypermethylation is generally observed [116,117] presumably to block the expression of stem cell suppressors. As differentiation progresses, higher and more general patterns of methylation emerge, particularly as a differentiated cell becomes more specialized in secretion patterns, like luminal cells in a normal prostate, and as described earlier for miRNA expression, which also functions as an expression repressor.

The ability of CpG methylations to control the expression of many genes in cancer has been extensively reviewed [62]. Glutathione S transferase (GSTP1) is an outstanding example of such methylation-driven control in the prostate [118,119], and genome-wide studies have been able to distinguish the methylation phenotypes of normal and malignant tissues and cells [120]. However, as the resolution of the analyses has improved, so have the subtleties associated with genomic hypo- and hyper-methylation. Firstly, methylation patterns in extracts of cancer biopsies are notoriously variable, certainly compared to cell cultures [121]. Tissue cell heterogeneity is a major influence on this, and has stimulated studies of cell-type-specific methylation in cells fractionated from tumors [122,123] in which many of the cancer-associated methylation patterns were reassigned to control of differentiation (i.e., a predominance of prostate luminal cells and loss of basal cells in cancer) and the replicative state of the previously terminally differentiated luminal cells. However, this was not an absolute rule, since oncogene activation has been reported through the loss of promoter DNA methylation [124]. Promoter hypomethylation in prostate cancer has also been reported to facilitate aberrant expression of Wnt5a affecting tumor malignancy [125], and the up-regulation of Wnt signaling by circulating tumor cells (CTCs) in CRPC patients [126].

Gene expression studies, closely coupled to genome-wide CpG methylations in prostate tissues have found that there was a relatively low correlation between the two phenomena, unlike similar studies in established cell lines [127]. In fractionated cell populations from prostate cancer tissues, we have recently identified sets of genes which displayed epigenetic control mediated by CpG methylations, but the strongest link remained with differentiated cell status: hence the apparent hypermethylation seen in cancers, with its predominantly (although aberrant) luminal phenotype [123].

**Figure 4:**
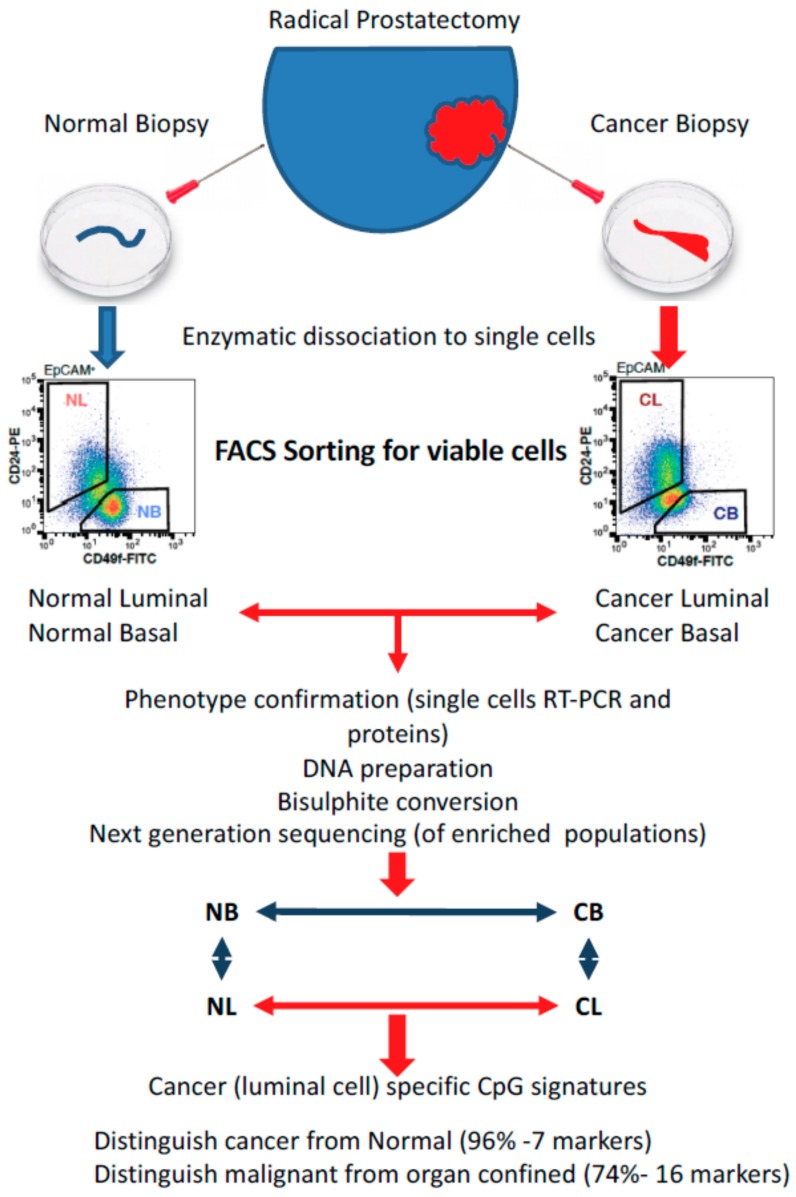
Fractionation strategy for genomic methylation analysis of discrete cell subpopulations from the prostate. Fractionation from total tissue biopsies as described in the text eliminates the influence of dominant cell populations and also eliminates artefacts of in vitro cell culture [128].

As a test of this hypothesis, we have recently carried out a genome-wide DNA sequencing of bisulfite-modified DNA extracted from multiple cell types, enriched according to their phenotype [128]. In contrast to all previous studies, the cells used had been directly enriched from disaggregated tissue biopsies of radical prostatectomies. In addition to a scan-directed intra-tumoral biopsy, we also obtained a normal tissue biopsy from a cancer-free location in the patient’s gland. The presence/absence of cancer in the tumor/normal needle cores was confirmed by subsequent histology. The procedure is outlined in Figure 4. After the methylated CpG sequences had been assigned to various loci, the closest association between patterns of methylation assigned different samples to the original patient, followed by the differentiated cell type of origin. Therefore, many of the existing genome-wide CpG methylation signatures for prostate cancer [120,129] will be contaminated with patient-specific signals and affected by the cellular composition of the biopsies used. To overcome this bias, the patient and cell-type specific signals were removed, allowing a direct comparison of non-malignant vs malignant epithelial populations in each patient. Here, very few CpG methylation changes were seen in the basal compartment: all of which were related to the gene ontology terms cell motility and cell adhesion—both essential properties of a cancer stem-like cell relative to its normal equivalent. However, for a comparison of normal and malignant tissues to be valid, and perhaps applicable in the clinic, normal luminal cells relative to the 99% of luminal-like cells in cancer biopsies are the most useful comparison. When this was carried out, again relatively few gene ontology terms were involved, but most notably CpG methylation was related to the luminal cell proliferation in cancer, relative to the terminally differentiated non-dividing status of normal luminal cells. 

Since these signatures had the cell and patient-specific elements removed, they were then applied to existing CpG methylation data in the TCGA prostate cancer database [115]. Unfortunately, the TCGA data were obtained from methylation oligonucleotide arrays, so the sequencing data was compressed into 100bp “bins” to match the two resources. This results in a loss of resolution. From TCGA data, the 553 patient analyses (of which 50 also had patient-matched normal tissues) contained 255 methylation array loci overlapping 1472 differentially methylated sequences. We were able to identify a small number of DMRs (differentially methylated regions) (8 × 100 bp sequences) which could distinguish normal from cancer luminal epithelium at a 96% efficiency. In fact, because the data eliminated patient and other cell type signatures, this assay should be able to find even small areas of cancer within normal tissues at the same efficiency.

Secondly, since the original tissues used to establish the signatures were from high Gleason grade samples, we next asked whether this signature was able to distinguish tumors with a poor outcome from those which remained organ-confined. Again, all of the relevant clinical outcomes were included in the TCGA information. By using 60% of the database information as a training set, we were able to devise a 17 DMR signature for patients whose cancers were likely to have spread. This resulted in a 76% detection rate for poorer outcomes, but from an original radical prostatectomy sample, i.e., at an earlier time before conventional technology such as MRI scanning could be used to locate micro-metastases. Thus, malignancy seems to be fixed in the CpG methylation patterns from an early time in cancer development. With more patients and matching of methylated CpG technologies (i.e., direct sequencing) this analysis could offer an early diagnosis of the most malignant prostate cancers.

The analysis also had a sounder mechanistic basis than an empirical serum PSA level increase. The cancer-related hypermethylated regions detected in the luminal compartments were normally in upstream enhancer regions, outside of CpG islands, Shores and Shelves [130]. As determined by reference to the ENCODE data, conserved enhancers more than 5 kb from transcriptional start sites [81] showed the most divergent CpG methylations. The DMRs were also marked by evolutionary conservation [131] the presence of “open” chromatin (detectable by DNAase I hypersensitivity) [132] and known transcription factor binding sites, previously found by genome-wide chromatin immunoprecipitation [133]. Expression of components of the polycomb complex such as EZH2 and SUZ12, previously shown to be overexpressed in prostate cancer [134,135], was controlled by CpG methylation, as were the transcription factor binding (TFB) sites in the upstream regions of TP63, TP53 and NF1. Hypomethylation was present in FOXA1, NFKB and GATA3 TFB sites in addition to a number of repetitive sequences collectively known as LINE and LTR but not SINE sequences, so hypomethylation was not simply a feature of nucleotide base repetition. Whilst established CpG methylation-controlled genes did show specific hypermethylation e.g., upstream of GSTP1 and CCDC8, in this case, it was apparent in the basal: luminal cell comparison and was located in the more widely studied promoter regions (CpG islands)—differentiation linked rather than associated with pathology [128].

## 14. Epigenetic Control of Random Mono-Allelic Gene Expression in Development and Cancer

Epigenetic control of gene expression is normally considered at the single gene level (e.g., CpG island methylation and GSTP1). However, most mammalian cells are diploid and prostate cancer cells are normally either quasi-diploid (hormone naïve) or aneuploid (CRPC), containing at least two copies of all non-sex chromosomes. One notable exclusion is the X-encoded androgen receptor with its critical role in PCa, although amplification of X and AR genes is seen in CRPC [64]. The selective silencing of one X chromosome in female cells [136] and persistence of gene expression from the maternal X chromosome in non-embryonic tissues during development [137] were prototypes for similar mono-allelic expression studies in the human autosomes.

High-resolution genome sequencing has now provided unprecedented details about the allelic nature of oncogene activation in prostate cancers, where most mutagenic changes are heterozygous, retaining a copy of the normal allele [115]. According to classical theory, all such oncogenic mutations should act in a dominant manner, and the activated gene product should be expressed in the cancer cells. However, selection of allelic preferences in multiple genes during mammalian development is controlled in a random fashion, unless it confers a selective advantage on a cell in which it expressed, overcoming the biological defenses against cancer development [138].

The presence of the normal allele is rarely considered when determining new potential therapeutic targeting and indeed understanding the cancer. As normal cells and tissues develop from a stem cell origin, virtually every cell inactivates one chromosomal copy by epigenetic mechanisms. Patches of clonally derived cells are also present in most tissues, for example, multiple cell patches containing inherited mitochondrial DNA mutations in the normal prostate [139].

An elegant single cell transcript analysis of allelic expression in developing mouse embryos showed mono-allelic expression of 12% to 24% of autosomal genes [140]. A generally stochastic pattern of monoallelic expression was seen, which differed from that in cells displaying allelic exclusion and genomic imprinting, whilst considerable variability was seen amongst closely related embryonic cells, and the phenomenon was also observed in more differentiated cells from mouse tissues [141]. Here, individual cells demonstrated a form of transcriptional bursting [142] independent surges of transcription could occur from both alleles over time in the same cell, but only RNA from a single allele was present at any given time.

## 15. Random Monoallelic Gene Expression in Human Cancers

Different cancer cells are likely to display variant allelic preferences between genes. Such Random Monoallelic Expression (RME) is distinct from expression controls established by aberrant promoter methylation, or locus deletion detected in cancers. In RME, the alleles of a specific gene can be (i) biallelically, (ii) maternal monoallelically, (iii) paternal monoallellically expressed or (iv) completely silenced. The expressed allele is stably retained after mitosis and differentiation in non-stem daughter cells [54,143]. From this point monoallelic expressing cells populate the tissue, creating an expression mosaic pattern based on initial allelic activation in the founding CSC. This mosaic expression pattern has been found in glioblastoma driver genes [144]. Whilst RME is most readily observed in tumor cell cultures [145], the contention that the phenomenon was an in vitro artefact has now been dismissed by in vivo analyses. [143,146].

In the majority of genes displaying RME, regulation of allelic choice is, however, not controlled by strand-specific methylation [54,144] of promoter CpG islands [147] as originally proposed [148]. High-resolution promoter analysis implies that RME is defined by an asymmetric chromatin signature in which histones within the gene body of an active allele are tri-methylated at H3K36 and the silenced allele methylated at H3K27 [146]. Regulation of allele-specific expression is therefore hardwired into chromatin, and many of the genes discovered in these screens were also present in bivalent chromatin loci, which is defined by dual deposition of activating (H3K4me3) and repressive (H3K27me3) histone “marks” at promoter regions, which is assumed to permit rapid activation of genes upon stem cell differentiation [149], as we have shown for prostate cancer stem-like cells [54]. Another pertinent example of allelic suppression is the haplo-insufficient expression of the PTEN gene in prostate cancers [150,151,152]. Whilst mutation/loss of both PTEN loci on chromosome 10 is common in CRPC, primary prostate cancers retain a normal allele, and PTEN is expressed in tissue extracts in low amounts [115,153]. RME would predict that complete silencing is seen in a proportion of cells, whereas in others the normal allele can be expressed at cytoprotective levels since homozygous knockouts result in high cancer rates but are also embryonic lethal [154].

Tumorigenic RME has been reported in peripheral mononuclear blood cells from chronic lymphocytic leukemia patients with DAPK1 haploinsufficiency [155], whereas in hepatocellular carcinomas, Illumina SNP chips identified 17 monoallelically expressed genes, 58 allelic genes with allelic imbalance, and seven genes showing allele substitution [156]. This silencing of activated oncogenes by RME has been reported in other common cancers, including colon and breast [157,158], but its effects are best illustrated in multiple myeloma, where only 27% of mutated genes (11%–47%) found in the cancer cells are actually expressed [159] in heterozygous cells, where the normal allele is preferentially transcribed.

The expression of TMPRSS2—which provides the promoter for the TMPRSS2-ERG fusion, presents in about 50% of organ-confined prostate cancers, the commonest genetic abnormality in prostate cancer, [10,53] is strongly regulated by RME. This perturbation of RME in prostate cancer is again the result of epigenetic control by histone code rather than by CpG methylation, generating epigenetic plasticity in the propagating cell, and resulting in expression heterogeneity [54].

Within a mass of identical cancer cells, epigenetic control of RME can both silence tumor suppressor loci and activate oncogenic alleles. However, it is now clear that tumors are not a homogeneous mass of identical cells. Pre-tumor progression, where mutations accumulate in a phenotypically silent manner has been proposed as the source of the multiple cooperating driver mutations in the final tumor. It is perhaps significant that treatment-naive prostate cancers have relatively few p53 mutations [160], to render the cells less susceptible to the “genomic protection” function of p53 and apoptotic death when a mutation is detected. However, this does not preclude epigenetic p53 silencing to permit cell replication and establishment of mutagenic changes. The same can be argued for changes to the immune suppression pathways, which normally result in the destruction of a cell expressing a neo-antigen. Allelic suppression as seen in RME would also suppress any endogenous immunosurveillance [161].

**Figure 5:**
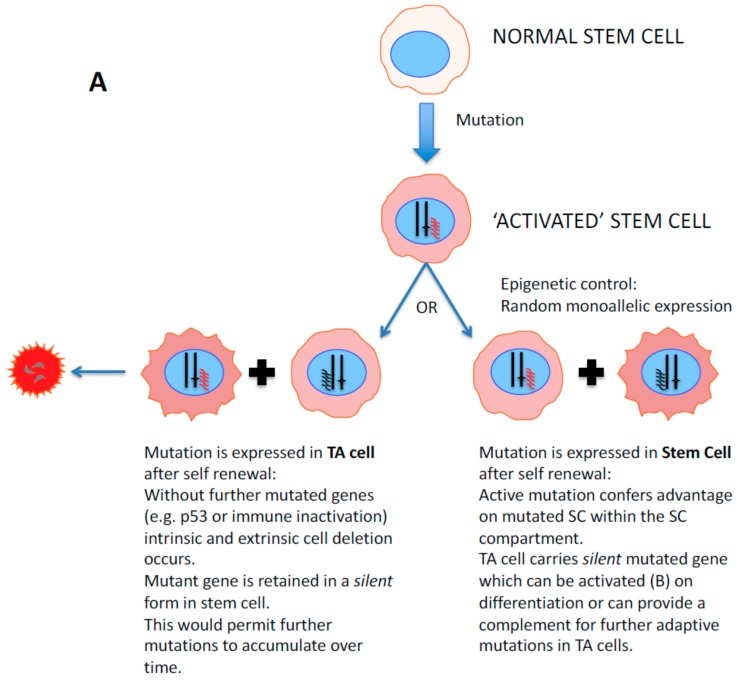

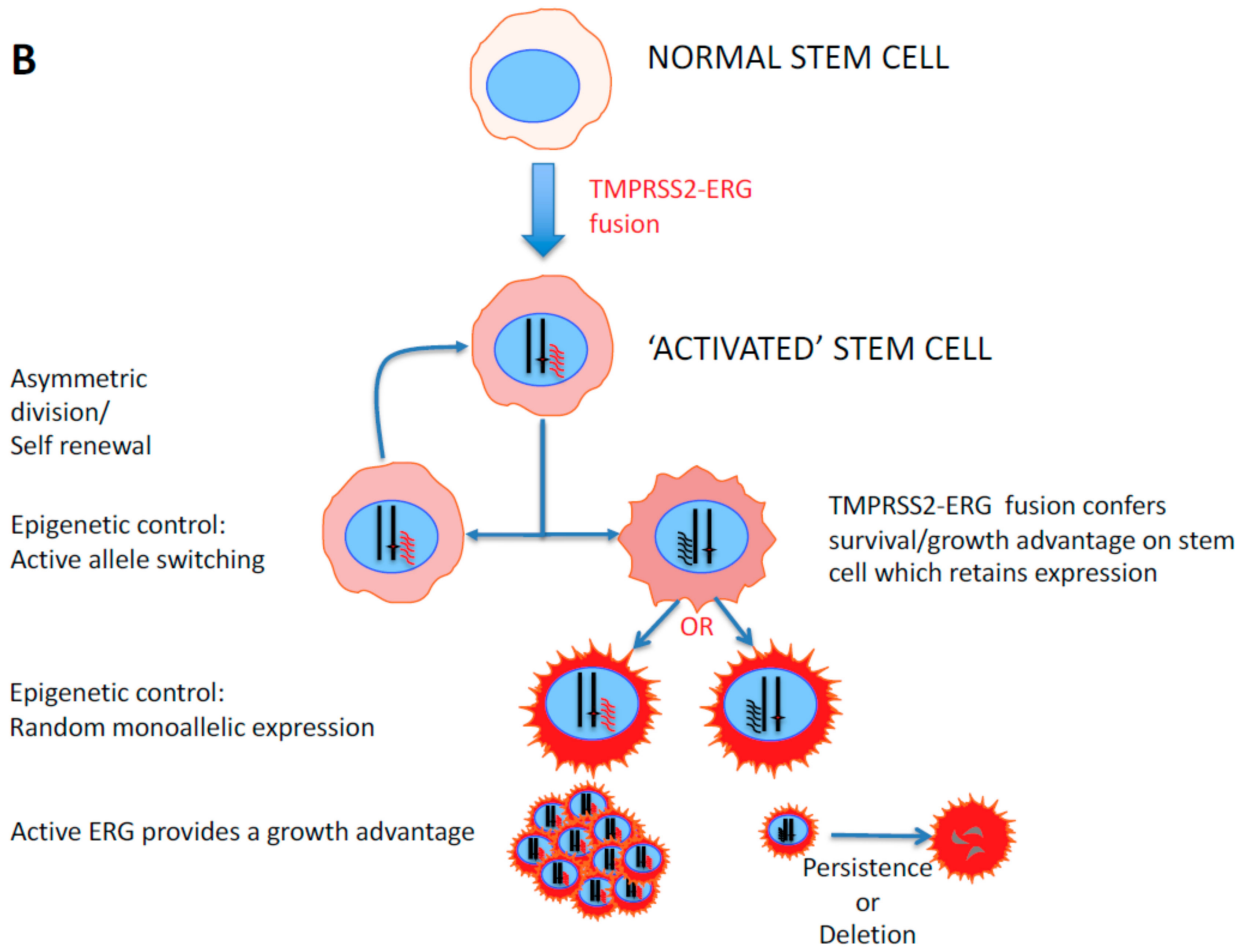
Epigenetic control of gene silencing and clonal deletion in prostate cancer development. (**A**) Activating mutations (or epigenetic controls) in the stem cell are either neutral (left) or confer a selective advantage for the SC. As shown on the left panel, in the absence of other changes the self-renewal results in an SC which does not express the activating copy, but rather the wild-type allele, whereas the TA cell does express the activating allele and may either expand or as illustrated for a single change, suffer intrinsic or extrinsic deletion. However, the SC remain as a pool of the heterozygous state, which can be subject to further changes during pre-tumor progression. The right panel shows the outcome when the activating gene *does* confer a selective advantage (like ERG in (**B**)). In this case, the TA cell retains the mutation, can expand and may restore expression of the mutant gene if RME is truly random, as we propose. (**B**) Epigenetic/allelic silencing of the TMPRSS2-ERG fusion gene. After asymmetric division (self-renewal) of the stem cells, the daughter cell population contains but does not express the fusion, whilst on further mitoses and differentiation the hyper-activated ERG (ETS transcription factor) gene is even more highly expressed, under androgen stimulation of the TMPRSS2 promoter [54]. This provides a selective growth advantage to ERG+ cells within the tumour mass.

The latter events are illustrated schematically in Figure 5A. Here the activating mutation (or epigenetic control) in the stem cell either is neutral (left) or confers a selective advantage for the SC (right). As shown on the left panel, in the absence of other changes, the self-renewal results in a SC which does not express the activated oncogene copy, but rather the wild-type allele, whereas the TA cell does express the activating allele and may either expand or as illustrated for a single change, suffer intrinsic or extrinsic deletion. However, the SC remains as a pool in the heterozygous state, which can be subject to further change in a pre-tumor progression [34]. The right panel shows the state when the activating gene does confer a selective advantage (like ERG in TMPRSS2-ERG fusions) [162,163]. In this case, the TA cell retains the mutation, can expand and may restore expression of the mutant gene if RME is truly random, as we suspect (Figure 5B). Hence clones of cells within the tumor, as seen in many immunohistochemical studies of prostate cancer, could ultimately emerge. This might also explain the increasing prevalence of “cancer mutations” in apparently normal tissues adjacent to tumor cells [164].

## 16. Conclusions: A Hypothesis for Epigenetic Control of Epithelial Cell Differentiation in Human Prostate

**Figure 6:**
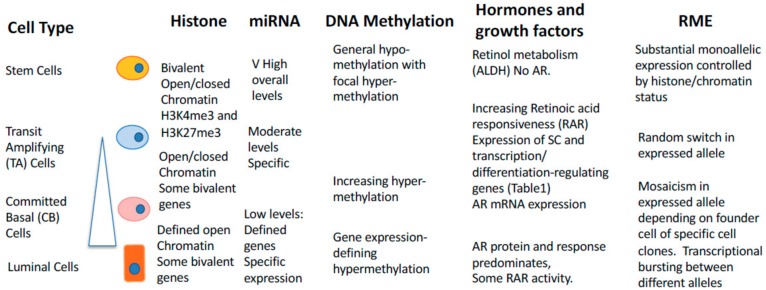
An integrated model of epigenetic control in prostate epithelium. The differentiation of prostate epithelium is controlled by multiple epigenetic influences. Between the defining self-renewal upon SC asymmetric cell division and the terminal differentiation into a luminal cell, there exists a continuum of differentiation (see shaded triangle). TA and CB cells are recognizable intermediates but exist within this continuum. As cells become more differentiated, the reversibility of the procedure becomes less likely (see Figure 1B). Details of individual controls are given in the text.

There is a prominent role for each of the major epigenetic control mechanisms in prostate epithelial cell differentiation. Histone modifications and changes to chromatin configuration provide the initial overall control of transcription and the selection of allelic preferences in gene expression. A cell stage-specific representation of this is shown in Figure 6. However, as stated earlier, there is a continuum of phenotypic changes between the defining asymmetric division of SC and the final and terminal differentiation into luminal cells. 

We have defined a set of transcription factors, including the critical Rock 2 kinase, which controls transcription of non-overlapping gene sets (Table 1 and Table 2). These master controllers are co-regulated not only in the prostate but in other human tissues. Hormonal and growth factors are clearly influential but act differentially on the individual cell types, for example, retinoids and glucocorticoids in SC, estrogen receptor in TA/CB cells and androgens in more luminal cells.

The SC state appears to be one of “active” quiescence, where expression at high levels of many miRNAs, and the existence of bivalent and/or poised chromatin (controlled by simultaneous binding of repressive and activating modified histones) indicates a cell which can react rapidly to changes in microenvironment/cell division, to produce a more differentiated daughter cell. Genomic methylation plays a less important, but not insignificant role, as SC are generally hypomethylated, with only a few clusters of hypermethylated chromatin. Some of these CpG clusters influence the SC adhesive properties i.e., sensing changes to the microenvironment, which may define fate after asymmetric division. However, the patterns of differential CpG methylation between normal and malignant cells from the same patient lie out-with the normally screened CpG “islands” can provide significant information about cellular processes involved in both carcinogenesis and differentiation, particularly if carried out on a patient-specific basis using matched normal and malignant tissues. 

In terms of understanding prostate cancer, and devising more effective and longer-lasting treatments, we need to consider the phenotype of not only the majority cell population within normal and malignant prostate epithelium but also minor populations such as progenitors and stem-like cells. The stem-like cells provide a strong argument in favor of an intrinsic therapy-resistant cell in cancers, rather than induction of resistance by the therapies. Since the ability to differentiate appears to be hard-wired- into all such stem-like cells, one solution would be to deplete or eliminate the SC by inducing differentiation, as shown in acute promyelocytic leukemia where pre-treatment with retinoic acid results in a more differentiated cell, which can be killed effectively by traditional chemotherapies.

Exploitation of this fundamental property of the resistant stem-like population i.e., a focus on cellular differentiation in prostate cancer, rather than the traditional description of cancer as a fast-growing cell, has now yielded three potentially novel strategies to improve treatment efficacy:

1. Treatment with inhibitors of histone deacetylase, (at a concentration about 100-fold lower than that used in cytotoxic cancer treatments) resulted in a 40% increase in SC radio-sensitivity, whilst not affecting the more differentiated and radiosensitive cells. The in vitro data strongly promotes the use of HDAC inhibitors (at sub-toxic doses) as radio-sensitizers in prostate cancer treatments.
2. Radiotherapy (and chemotherapy) patients are often treated with glucocorticoids to counteract the side-effects of treatment. In our studies of miR99a/100 in primary PCa, pre-treatment with dexamethasone stimulated miR99a/100, reducing SMARC expression and decreased radiotherapy responses, whereas combination treatment with the GCR inhibitor Mifepristone increased radio-sensitivity by stimulating the expression levels of miR-99a/100 and decreasing SMARC-induced chromatin condensation. Therefore, the clinical use of GCR inhibitors should clinically enhance radiotherapy and perhaps reduce tumor relapse.
3. Overexpression of exogenous miR-548c-3p, which is highly expressed in PCa SC, made radiosensitive CB cells more resistant to irradiation, by induction of a more stem-like state. Thus, inhibition of the SC-preserving activity of high miR-548c-3p levels should also increase clinical radiotherapy efficacy.

## 17. Future Perspectives and Challenges

**Figure 7:**
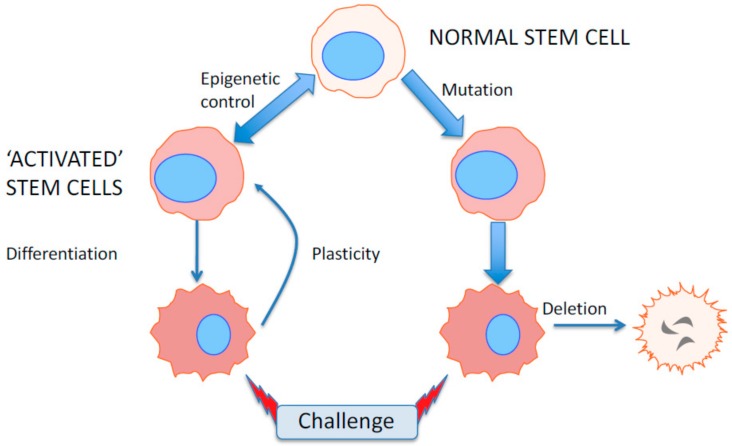
Epigenetic control of gene silencing and clonal deletion in prostate cancer development. Epigenetic gene activation/repression allows the activated stem cells in cancer or pre-cancer to revert to a resistant phenotype when challenged with extrinsic or intrinsic mutant cell deletion whereas mutational activation cannot be reversed, allowing the cells to be deleted.

To paraphrase what Waddington so beautifully illustrated almost 80 years ago: by getting stem-like cells in cancer to roll down into epigenetic valleys, we can perhaps kill more of them by conventional means. The challenge faced by researchers wishing to translate similar findings into clinical practice is the similarity between the epigenetic control of normal and malignant cell differentiation. Preservation of normal stem cell function, not only in the target organ, but elsewhere in the body remains difficult to predict, and off-target effects may take some time to manifest themselves if affecting slowly cycling stem cells. A major feature of Waddington’s Hypothesis was the prediction of developmental plasticity mediated by epigenetic control. Mutational changes to the cancer genome are essentially irreversible and rather stupid evolutionary strategies and are mainly exploited by prostate cancers as a response to therapy. A smart tumor cell will maintain its options in a changing tumor microenvironment (Figure 7).

Finally, the data from prostate and other cancers provides insights to be gained from a cell fractionation approach to complex tissues, prior to deep sequencing or similar analyses, and its ability to generate genuinely novel targets for cancer therapy. Recent data also clearly indicate the compromise offered by analysis of established cell lines, which have been selected for their ability to grow (sometimes more than 40 years ago), compared to tissues. The high quantity, high heterogeneity, high depth approaches to the analysis of tissues also present problems with interpretation—requiring large numbers of cells. Rather, current efforts to miniaturize key assays such as genomic methylation, chromatin immunoprecipitation, and better fidelity DNA/RNA sequencing from single cells are all key to the study of minor cell populations.

With major pharmaceutical companies seemingly focused on generating biosimilar drugs in a restricted pool of therapeutics, offering life extensions of months and at a high cost in many cases, relative to either standard of care or placebo treatments, the ability to kill more of the disparate cell types in a tumor based on epigenetic mechanisms, in a “low mutation” primary cancer such as prostatic adenocarcinomas.

## Figures and Tables

**Table 1:** Co-regulated gene clusters during prostate epithelial cell differentiation.

Gene Group	Common Gene Ontology Terms	Selected Members
A	None	PSEN1 ITGB6 IRF6
B	Cell cycle Chromatin condensation	CDCA2 TOP2A CDC20
C	Epidermal differentiation Endopeptidase activity	TMPRSS2 S100P SPINK7 ELF3 LXN MSMB
D	(Lens) Epithelial development	CTNNB1 IGFR2

Co-regulated genes expressed as discrete non-overlapping clusters (Figure 2A) during prostate epithelial cell differentiation. Data from Reference [26].

**Table 2:** Major transcription factors implicated in prostate epithelial cell differentiation.

TF Identity	Full Name	Principal Role
RXR	Retinoid X receptor: acts as a homodimer or as a heterodimer with other receptors (e.g., VDR). Binds co-repressors of transcription (as a repressor) until a conformation change occurs after ligand binding.	Reproduction, cellular differentiation, bone development, haematopoiesis and pattern formation during embryogenesis
VDR	Vitamin D Receptor: homodimer in the absence of ligand then heterodimerises with RXR to increase transcription of a number of genes. Interacts with SMAD3 and MED1, NCOA1, NCOA2, NCOA3 and NCOA6 coactivators.	Mineral metabolism (calcium homeostasis) although VDR regulates a variety of other pathways, such as those involved in the immune response and cancer. Keratinocyte, mammary and prostate differentiation.
GCR	Glucocorticoid Receptor (N3CR1): acts both as a transcription factor and modulator of other transcription factors by binding to glucocorticoid response elements (GRE), both in the cell nucleus and mitochondria.	Affects inflammatory responses, cellular proliferation and differentiation in target tissues. Also involved in chromatin remodeling and RNA stability/degradation.
TAZ	Transcriptional co-activator with PDZ-binding motif (or WWTR1): acts as a transcriptional co-activator, downstream of the Hippo pathway. Regulated by soluble extra-cellular factors, cell–cell adhesions and mechano-transduction. Interacts with and regulates multiple transcription factors, e.g., Runx2 PPAR TBX5, TBX5, TEADs, TTF-1 and PAX3.	Organ development, stem cell differentiation and development of human cancer. Mesenchymal stem cell differentiation, promoting cell proliferation and epithelial-mesenchymal transition (EMT). TAZ senses different cellular signals such as cell density and the extracellular matrix stiffness. Significantly overexpressed in breast cancer samples and papillary thyroid carcinoma tissues.
SRF	Serum Response Factor: member of the MADS box superfamily of transcription factors, and binds to the serum response element (SRE) in the promoter region of target genes. SRF regulates the activity of many immediate-early genes, e.g., c-fos. A downstream target of many pathways; for example, the mitogen-activated protein kinase pathway (MAPK).	Stimulates cell cycle regulation, apoptosis, cell growth, and cell differentiation. In embryonic development, Expression controls the formation of mesoderm and is crucial for the growth of skeletal muscle. Interaction of SRF with other proteins, such as steroid hormone receptors, may contribute to the regulation of muscle growth by steroids.
HSF1	Heat shock transcription factor 1: an inactive monomer in a complex with Hsp40/Hsp70 and Hsp90. Target genes include major inducible heat shock proteins such as Hsp72 and noncoding RNA within Satellite III repeat regions. Upon stress, such as elevated temperature, HSF1 is released from the chaperone complex and trimerizes. HSF1 is then transported into the nucleus where it is hyperphosphorylated and binds to heat shock elements in DNA.	Master regulator of stress responses, mammalian development, insulin metabolism, cell division, transcriptional reprogramming/chromatin status.
ROCK2	Rho-associated coiled coil-containing protein kinase 2: regulates smooth muscle contraction, actin cytoskeleton organization, stress fiber and focal adhesion formation, neurite retraction, cell adhesion and motility via phosphorylation of ADD1, BRCA2, CNN1, EZR, DPYSL2, EP300, MSN, MYL9/MLC2, NPM1, RDX, PPP1R12A and VIM. Phosphorylates SORL1 and IRF4. Acts as a negative regulator of VEGF-induced angiogenic endothelial cell activation and inhibits keratinocyte terminal differentiation.	Regulates cytoplasmic actin and cell polarity. Major regulator of epithelial terminal differentiation.

Major transcriptional effectors of the stem cell state identified in promoter analyses of differentially regulated genes matched to their known expression levels in prostate epithelial stem cells. Data from Reference [26].

**Table 3:** Principal miRNA classes implicated in prostate epithelial cell differentiation.

SC Signature	Specific PCa CSC Signature	Specific CRPC CSC Signature
**Upregulated miRNA**		
miR-302 family	miR-33a *	let-7i *
miR-371 family	miR-181a-2 *	miR-136
miR-484	miR-323-3p	miR-143
miR-548c-3p	miR-411 *	miR-214 *
	miR-487b	miR-362-5p
	miR-532-3p	miR-516a-5p
	miR-1271	miR-542-5p
		miR-545
		miR-1913
**Downregulated miRNA**		
let-7 family	miR-302c	miR-125b-2 *
miR-8 family	miR-519c-3p	miR-708
miR-10 family	miR-574-5p	
miR-17-92 family	miR-1181	
miR-99a/100		
miR-143		
miR-145		

Upregulated miRNA indicated in green; Downregulated miRNA in red. CRPC = castration-resistant prostate cancer; CSC = cancer stem-like cell; PCa = prostate cancer; SC = stem cell. * Lower prevalence product of a specific miRNA locus. Data taken from References [27,28].

## References

[1] Packer J.R., Maitland N.J. (2016). The Molecular and Cellular Origin of Human Prostate Cancer. Biochim. Biophys. Acta.

[2] Tannock I.F., de Wit R., Berry W.R., Horti J., Pluzanska A., Chi K.N., Oudard S., Theodore C., James N.D., Turesson I. (2004). Docetaxel Plus Prednisone or Mitoxantrone Plus Prednisone for Advanced Prostate Cancer. N. Engl. J. Med..

[3] Gleason D.F. (1966). Classification of prostatic carcinomas. Cancer Chemother. Rep..

[4] MacIntosh A.C., Stower M., Reid N., Maitland N.J. (1998). Precise microdissection of human prostate cancers reveals genotypic heterogeneity. Cancer Res..

[5] Beltran H., Prandi D., Mosquera J.M., Benelli M., Puca L., Cyrta J., Marotz C., Giannopoulou E., Chakravarthi B.V., Varambally S. (2016). Divergent clonal evolution of castration-resistant neuroendocrine prostate cancer. Nat. Med..

[6] Hayward S.W., Wang Y., Cao M., Hom Y.K., Zhang B., Grossfeld G.D., Sudilovsky D., Cunha G.R. (2001). Malignant transformation in a nontumorigenic human prostatic epithelial cell line. Cancer Res..

[7] Hall A.J., Maitland N.J., Stower M., Lang S.H. (2002). Primary prostate stromal cells modulate the morphology and migration of primary prostate epithelial cells in type 1 collagen gels. Cancer Res..

[8] Mo F., Lin D., Takhar M., Ramnarine V.R., Dong X., Bell R.H., Volik S.V., Wang K., Xue H., Wang Y. (2018). Stromal Gene Expression Is Predictive for Metastatic Primary Prostate Cancer. Eur. Urol..

[9] Dunne P.D., McArt D.G., Bradley C.A., O’Reilly P.G., Barrett H.L., Cummins R., O’Grady T., Arthur K., Loughrey M., Allen W.L. (2016). Challenging the cancer molecular stratification dogma: Intratumoral heterogeneity undermines consensus molecular subtypes and potential diagnostic value in colorectal cancer. Clin. Cancer Res..

[10] Berger M.F., Lawrence M.S., Demichelis F., Drier Y., Cibulskis K., Sivachenko A.Y., Sboner A., Esgueva R., Pflueger D., Sougnez C. (2011). The Genomic Complexity of Primary Human Prostate Cancer. Nature.

[11] Grasso C.S., Wu Y.-M., Robinson R., Cao X., Dhanasekaran S.M., Khan A.P., Quist M.J., Jing X., Lonigro R.J., Brenner J.C. (2013). The Mutational Landscape of Lethal Castration-Resistant Prostate Cancer. Nature.

[12] Sharifi N. (2013). Mechanisms of Androgen Receptor Activation in Castration-Resistant Prostate Cancer. Endocrinology.

[13] Chandrasekar T., Yang J.C., Gao A.C., Evans C.P. (2015). Mechanisms of Resistance in Castration-Resistant Prostate Cancer (CRPC). Transl. Androl. Urol..

[14] Goldstein A., Toro P.V., Lee J., Silberstein J.L., Nakazawa M., Waters I., Cravero K., Chu D., Cochran R.L., Kim M. (2017). Detection Fidelity of AR Mutations in Plasma Derived Cell-Free DNA. Oncotarget.

[15] Endrullat C., Glökler J., Franke P., Frohme M. (2016). Standardization and Quality Management in Next-Generation Sequencing. Appl. Transl. Genomics.

[16] Darmanis S., Sloan S.A., Croote D., Mignardi M. (2017). Single-Cell RNA-Seq Analysis of Infiltrating Neoplastic Cells at the Migrating Front of Human Glioblastoma. Cell Rep..

[17] Zeisel A., M͡oz-Manchado A.B., Codeluppi S., Lönnerberg P., Manno G.L., Juréus A., Marques S., Munguba H., He L., Betsholtz C. (2015). Cell Types in the Mouse Cortex and Hippocampus Revealed by Single-Cell RNA-Seq. Science.

[18] Tirosh I., Izar B., Prakadan S.M., Wadsworth M.H., Treacy D., Trombetta J.J., Rotem A., Rodman C., Lian C., Murphy G. (2016). Dissecting the Multicellular Ecosystem of Metastatic Melanoma by Single-Cell RNA-Seq. Science.

[19] Williams M.J., Werner B., Barnes C.P., Graham T.A., Sottoriva A. (2016). Identification of Neutral Tumor Evolution Across Cancer Types. Nat. Genet..

[20] Baca S.C., Prandi D., Lawrence M.S., Mosquera J.M., Romanel A., Drier Y., Park K., Kitabayashi N., MacDonald T.Y., Ghandi M. (2013). Punctuated Evolution of Prostate Cancer Genomes. Cell.

[21] Barbieri C.E., Baca S.C., Lawrence M.S., Demichelis F., Blattner M., Theurillat J.-P., White T.A., Stojanov P., Van Allen E., Stransky N. (2012). Exome Sequencing Identifies Recurrent SPOP, FOXA1 and MED12 Mutations in Prostate Cancer. Nat. Genet..

[22] An J., Wang C., Deng Y., Yu L., Huang H. (2014). Destruction of Full-LengthAndrogen Receptor by Wild-Type SPOP, but Not Prostate-Cancer-Associated Mutants. Cell Rep..

[23] Ross R.W., Galsky M.D., Scher H.I., Magidson J., Wassmann K., Lee G.-S.M., Katz L., Subudhi S.K., Anand A., Fleisher M. (2012). A Whole-Blood RNA Transcript-Based Prognostic Model in Men with Castration-Resistant Prostate Cancer: A Prospective Study. Lancet Oncol..

[24] Olmos D., Brewer D., Clark J., Danila D.C., Parker C., Attard G., Fleisher M., Reid A.H., Castro E., Sandhu S.K. (2012). Prognostic Value of Blood mRNA Expression Signatures in Castration-Resistant Prostate Cancer: A Prospective, Two-Stage Study. Lancet Oncol..

[25] Bonnet D., Dick J.E. (1997). Human Acute Myeloid Leukemia Is Organized as a Hierarchy That Originates From a Primitive Hematopoietic Cell. Nat. Med..

[26] Rane J.K., Droop A.P., Pellacani D., Polson E.S., Simms M.S., Collins A.T., Caves L.S.D., Maitland N.J. (2014). Conserved Two-Step Regulatory Mechanism of Human Epithelial Differentiation. Stem Cell Rep..

[27] Rane J.K., Scaravilli M., Ylipää A., Pellacani D., Mann V.M., Simms M.S., Nykter M., Collins A.T., Visakorpi T., Maitland N.J. (2015). MicroRNA Expression Profile of Primary Prostate Cancer Stem Cells as a Source of Biomarkers and Therapeutic Targets. Eur. Urol..

[28] Rane J.K., Ylipaa A., Adamson R., Mann V.M., Simms M.S., Collins A.T., Visakorpi T., Nykter M., Maitland N.J. (2015). Construction of Therapeutically Relevant Human Prostate Epithelial Fate Map by Utilising miRNA and mRNA Microarray Expression Data. Br. J. Cancer.

[29] Maitland N.J., Frame F.M., Polson E.S., Lewis J.L., Collins A.T. (2011). Prostate Cancer Stem Cells: Do They Have a Basal or Luminal Phenotype?. Hormones Cancer.

[30] Goldstein A.S., Huang J., Guo C., Garraway I.P., Witte O.N. (2010). Identification of a Cell of Origin for Human Prostate Cancer. Science.

[31] Lawson D.A., Zong Y., Memarzadeh S., Xin L., Huang J., Witte O.N. (2010). Basal Epithelial Stem Cells Are Efficient Targets for Prostate Cancer Initiation. Proc. Natl. Acad. Sci. USA.

[32] Wang X.I., Kruithof-de Julio M., Economides K.D., Walker D., Yu H., Halili M.V., Hu Y., Price S.M., Abate-Shen C., Shen M.M. (2009). A Luminal Epithelial Stem Cell That Is a Cell of Origin for Prostate Cancer. Nature.

[33] Tomasetti C., Vogelstein B. (2015). Cancer Etiology. Variation in Cancer Risk Among Tissues Can Be Explained by the Number of Stem Cell Divisions. Science.

[34] Calabrese P., Tavaré S., Shibata D. (2004). Pretumor Progression: Clonal Evolution of Human Stem Cell Populations. Am. J. Pathol..

[35] Reinhardt H.C., Schumacher B. (2012). The P53 Network: Cellular and Systemic DNA Damage Responses in Aging and Cancer. Trends Genet. TIG.

[36] Victorelli S., Passos J.F. (2017). Telomeres and Cell Senescence—Size Matters Not. EBioMedicine.

[37] Nonn L., Ananthanarayanan V., Gann P.H. (2009). Evidence for Field Cancerization of the Prostate. Prostate.

[38] Mehrotra J., Varde S., Wang H., Chiu H., Vargo J., Gray K., Nagle R.B., Neri J.R., Mazumder A. (2008). Quantitative, Spatial Resolution of the Epigenetic Field Effect in Prostate Cancer. Prostate.

[39] Slaughter P., Southwick H.W., Smejkal W. (1953). Field Cancerization in Oral Stratified Squamous Epithelium; Clinical Implications of Multicentric Origin. Cancer.

[40] Gundem G., Van Loo P., Kremeyer B., Alexandrov L.B., Tubio J.M.C., Papaemmanuil E., Brewer D.S., Kallio H.M.L., Hognas G., Annala M. (2015). The Evolutionary History of Lethal Metastatic Prostate Cancer. Nature.

[41] Birnie R., Bryce S.D., Roome C., Dussupt V., Droop A., Lang S.H., Berry P.A., Hyde C.F., Lewis J.L., Stower M.J. (2008). Gene Expression Profiling of Human Prostate Cancer Stem Cells Reveals a Pro-Inflammatory Phenotype and the Importance of Extracellular Matrix Interactions. Genome Biol..

[42] Collins A.T., Berry P.A., Hyde C., Stower M.J., Maitland N.J. (2005). Prospective Identification of Tumorigenic Prostate Cancer Stem Cells. Cancer Res..

[43] Frame F.M., Pellacani D., Collins A.T., Maitland N.J. (2016). Harvesting Human Prostate Tissue Material and Culturing Primary Prostate Epithelial Cells. Methods Mol. Biol..

[44] Vezina C.M., Allgeier S.H., Fritz W.A., Moore R.W., Strerath M., Bushman W., Peterson R.E. (2008). Retinoic Acid Induces Prostatic Bud Formation. Dev. Dyn..

[45] Oldridge E.E., Walker H.F., Stower M.J., Simms M.S., Mann V.M., Collins A.T., Pellacani D., Maitland N.J. (2013). Retinoic Acid Represses Invasion and Stem Cell Phenotype by Induction of the Metastasis Suppressors RARRES1 and LXN. Oncogenesis.

[46] Waltregny D., Leav I., Signoretti S., Soung P., Lin D., Merk F., Adams J.Y., Bhattacharya N., Cirenei N., Loda M. (2001). Androgen-Driven Prostate Epithelial Cell Proliferation and Differentiation in Vivo Involve the Regulation of P27. Mol. Endocrinol..

[47] Thomas M.A., Hodgson M.C., Loermans S.D., Hooper J., Endersby R., Bentel J.M. (2006). Transcriptional Regulation of the Homeobox Gene NKX3.1 by All-Trans Retinoic Acid in Prostate Cancer Cells. J. Cell. Biochem..

[48] Rane J.K., Pellacani D., Maitland N.J. (2012). Advanced Prostate Cancer—A Case for Adjuvant Differentiation Therapy. Nat. Rev. Urol..

[49] Goossens C., Deboel L., Swinnen J.V., Roskams T., Manin M., Rombauts W., Verhoeven G. (2002). Both Retinoids and Androgens Are Required to Maintain or Promote Functional Differentiation in Reaggregation Cultures of Human Prostate Epithelial Cells. Prostate.

[50] Rivera-Gonzalez G.C., Droop A.P., Rippon H.J., Tiemann K., Pellacani D., Georgopoulos L.J., Maitland N.J. (2012). Retinoic Acid and Androgen Receptors Combine to Achieve Tissue Specific Control of Human Prostatic Transglutaminase Expression: A Novel Regulatory Network with Broader Significance. Nucleic Acids Res..

[51] Zhang C., Zhang S., Zhang Z., He J., Xu Y., Liu S. (2014). ROCK Has a Crucial Role in Regulating Prostate Tumor Growth Through Interaction with C-Myc. Oncogene.

[52] Claassen D.A., Desler M.M., Rizzino A. (2009). ROCK Inhibition Enhances the Recovery and Growth of Cryopreserved Human Embryonic Stem Cells and Human Induced Pluripotent Stem Cells. Mol. Reprod. Dev..

[53] Tomlins S.A., Rhodes D.R., Perner S., Dhanasekaran S.M., Mehra R., Sun X.W., Varambally S., Cao X., Tchinda J., Kuefer R. (2005). Recurrent Fusion of TMPRSS2 and ETS Transcription Factor Genes in Prostate Cancer. Science.

[54] Polson E.S., Lewis J., Celik H., Mann V.M., Stower M.J., Simms M.S., Rodrigues G., Collins A.T., Maitland N.J. (2013). Monoallelic Expression of TMPRSS2/ERG in Prostate Cancer Stem Cells. Nat. Commun..

[55] Frame F.M., Maitland N.J., Cramer S.D. (2013). Cancer Stem Cells Provide New Insights into the Therapeutic Responses of Human Prostate Cancer. Stem Cells and Prostate Cancer.

[56] Petrie K., Zelent A., Waxman S. (2009). Differentiation Therapy of Acute Myeloid Leukemia: Past, Present and Future. Curr. Opin. Hematol..

[57] Lin P.-C., Hsieh H.-Y., Chu P.-C., Chen C.S. (2018). Therapeutic Opportunities of Targeting Histone Deacetylase Isoforms to Eradicate Cancer Stem Cells. Int. J. Mol. Sci..

[58] Salvador M.A., Wicinski J., Cabaud O., Toiron Y., Finetti P.J., Lelièvre H., Kraus-Berthier L., Depil S., Bertucci F., Collette Y. (2013). The Histone Deacetylase Inhibitor Abexinostat Induces Cancer Stem Cells Differentiation in Breast Cancer with Low Xist Expression. Clin. Cancer Res..

[59] Waddington C.M. (1940). Organisers and Genes.

[60] Papp B., Plath K. (2013). Epigenetics of Reprogramming to Induced Pluripotency. Cell.

[61] Aranda-Anzaldo A., Dent M.A.R. (2018). Landscaping the Epigenetic Landscape of Cancer. Prog. Biophys. Mol. Biol..

[62] Feinberg A.P., Koldobskiy M.A., Göndör A. (2016). Epigenetic Modulators, Modifiers and Mediators in Cancer Aetiology and Progression. Nat. Rev. Genet..

[63] McDonald O.G., Li X., Saunders T., Tryggvadottir R., Mentch S.J., Warmoes M.O., Word A.E., Carrer A., Salz T.H., Natsume S. (2017). Epigenomic Reprogramming During Pancreatic Cancer Progression Links Anabolic Glucose Metabolism to Distant Metastasis. Nat. Genet..

[64] Visakorpi T., Hyytinen E., Koivisto P., Tanner M., Keinänen R., Palmberg C., Palotie A., Tammela T., Isola J., Kallioniemi O.P. (1995). In Vivo Amplification of the Androgen Receptor Gene and Progression of Human Prostate Cancer. Nat. Genet..

[65] Horns R.C., Dower W.J., Schimke R.T. (1984). Gene Amplification in a Leukemic Patient Treated with Methotrexate. J. Clin. Oncol..

[66] Feinberg A.P., Ohlsson R., Henikoff S. (2006). The Epigenetic Progenitor Origin of Human Cancer. Nat. Rev. Genet..

[67] Przyborski S., Carr-Wilkinson J., Robson C.N., Heer R. (2013). A Novel Model of Urinary Tract Differentiation, Tissue Regeneration, and Disease: Reprogramming Human Prostate and Bladder Cells into Induced Pluripotent Stem Cells. Eur. Urol..

[68] Maitland N.J., Tindall D.J. (2013). Stem Cells in the Normal and Malignant Prostate. Prostate Cancer.

[69] Neilson J.R., Zheng G.X.Y., Burge C.B., Sharp P.A. (2007). Dynamic Regulation of miRNA Expression in Ordered Stages of Cellular Development. Genes Dev..

[70] Mathieu J., Ruohola-Baker H. (2013). Regulation of Stem Cell Populations by microRNAs. Transcript. Transl. Regul. Stem Cells.

[71] Catto J.W.F., Alcaraz A., Bjartell A.S., De Vere White R., Evans C.P., Fussel S., Hamdy F.C., Kallioniemi O., Mengual L., Schlomm T. (2011). MicroRNA in Prostate, Bladder, and Kidney Cancer: A Systematic Review. Eur. Urol..

[72] Zoni E., Van Der Horst G., van de Merbel A.F., Chen L., Rane J.K., Pelger R.C.M., Collins A.T., Visakorpi T., Snaar-Jagalska B.E., Maitland N.J. (2015). miR-25 Modulates Invasiveness and Dissemination of Human Prostate Cancer Cells via Regulation of Av- and A6-Integrin Expression. Cancer Res..

[73] Liu C., Kelnar K., Vlassov A.V., Brown D., Wang J., Tang D.G. (2012). Distinct microRNA Expression Profiles in Prostate Cancer Stem/Progenitor Cells and Tumor-Suppressive Functions of Let-7. Cancer Res..

[74] Leonardo T.R., Schultheisz H.L., Loring J.F. (2012). The Functions of microRNAs in Pluripotency and Reprogramming. Nat. Cell Biol..

[75] Jalava S.E., Urbanucci A., Latonen L., Waltering K.K. (2012). Androgen-Regulated miR-32 Targets BTG2 and Is Overexpressed in Castration-Resistant Prostate Cancer. Oncogene.

[76] Taylor B.S., Schultz N., Hieronymus H., Gopalan A., Xiao Y., Carver S., Arora V.K., Kaushik P., Cerami E., Reva B. (2010). Integrative Genomic Profiling of Human Prostate Cancer. Cancer Cell.

[77] Sadeghi M., Ranjbar B., Ganjalikhany M.R., Khan F.M., Schmitz U., Wolkenhauer O., Gupta S.K. (2016). MicroRNA and Transcription Factor Gene Regulatory Network Analysis Reveals Key Regulatory Elements Associated with Prostate Cancer Progression. PLoS ONE.

[78] Formosa A., Markert E.K., Lena A.M., Italiano D.O. (2014). MicroRNAs, miR-154, miR-299-5p, miR-376a, miR-376c, miR-377, miR-381, miR-487b, miR-485-3p, miR-495 and miR-654-3p, Mapped to the 14q32. 31 Locus regulate proliferation, apoptosis, migration and invasion in metastatic prostate cancer cells. Oncogene.

[79] Saini S., Majid S., Shahryari V., Arora S., Yamamura S., Chang I., Zaman M.S., Deng G., Tanaka Y., Dahiya R. (2012). miRNA-708 Control of CD44+ Prostate Cancer-Initiating Cells. Cancer Res..

[80] Josson S., Lao K., Chung L.W., Johnstone P.A., Sung S.-Y., Sung S. (2008). Radiation modulation of microRNA in prostate cancer cell lines. Prostate.

[81] Park Y.M., Cheong H.S., Lee J.-K. (2014). Genome-Wide Detection of Allelic Gene Expression in Hepatocellular Carcinoma Cells Using a Human Exome SNP Chip. Gene.

[82] Chivukula R.R., Shi G., Acharya A., Mills E.W., Zeitels L.R., Anandam J.L., Abdelnaby A.A., Balch G.C., Mansour J.C., Yopp A.C. (2014). An Essential Mesenchymal Function for miR-143/145 in Intestinal Epithelial Regeneration. Cell.

[83] Peng X., Guo W., Liu T., Wang X., Tu X., Xiong D., Chen S., Lai Y., Du H., Chen G. (2011). Identification of miRs-143 and -145 That Is Associated with Bone Metastasis of Prostate Cancer and Involved in the Regulation of EMT. PloS ONE.

[84] Lee H.K., Hsu A.K., Sajdak J., Qin J., Pavlidis P. (2004). Coexpression Analysis of Human Genes Across Many Microarray Data Sets. Genome Res..

[85] Turdo A., Veschi V., Gaggianesi M., Chinnici A., Bianca P., Todaro M., Stassi G. (2019). Meeting the Challenge of Targeting Cancer Stem Cells. Front. Cell Dev. Biol..

[86] Skvortsov S., Debbage P., Lukas P., Skvortsova I. (2015). Crosstalk Between DNA Repair and Cancer Stem Cell (CSC) Associated Intracellular Pathways. Semin. Cancer Biol..

[87] Skvortsova I., Debbage P., Kumar V., Skvortsov S. (2015). Radiation Resistance: Cancer Stem Cells (CSCs) and Their Enigmatic Pro-Survival Signaling. Semin. Cancer Biol..

[88] Lukas J., Lukas C., Bartek J. (2011). More Than Just a Focus: The Chromatin Response to DNA Damage and Its Role in Genome Integrity Maintenance. Nat. Cell Biol..

[89] Takata H., Hanafusa T., Mori T., Shimura M., Iida Y., Ishikawa K., Yoshikawa K., Yoshikawa Y., Maeshima K. (2013). Chromatin Compaction Protects Genomic DNA From Radiation Damage. Edited by Yamini Dalal. PLoS ONE.

[90] Falk M., Lukášová E., Kozubek S. (2008). Chromatin Structure Influences the Sensitivity of DNA to Γ-Radiation. Biochim. Biophys. Acta.

[91] Srikantan S., Abdelmohsen K., Lee E.K., Tominaga K., Subaran S.S., Kuwano Y., Kulshrestha R., Panchakshari R., Kim H.H., Yang X. (2011). Translational Control of TOP2A Influences Doxorubicin Efficacy. Mol. Cell. Biol..

[92] Nguyen H.C.N., Xie W., Yang M., Hsieh C.-L., Drouin S., Lee G.-S.M., Kantoff P.W. (2013). Expression Differences of Circulating microRNAs in Metastatic Castration Resistant Prostate Cancer and Low-Risk, Localized Prostate Cancer. Prostate.

[93] Hall A.W., Battenhouse A.M., Shivram H., Morris A.R., Cowperthwaite M.C., Shpak M., Iyer V.R. (2018). Bivalent Chromatin Domains in Glioblastoma Reveal a Subtype-Specific Signature of Glioma Stem Cells. Cancer Res..

[94] Voigt P., Tee W.W., Reinberg D. (2013). A Double Take on Bivalent Promoters. Genes Dev..

[95] Bernstein B.E., Mikkelsen T.S., Xie X., Kamal M. (2006). A Bivalent Chromatin Structure Marks Key Developmental Genes in Embryonic Stem Cells. Cell.

[96] Li F., Wan M., Zhang B., Peng Y., Zhou Y., Pi C., Xu X., Ye L., Zhou X., Zheng L. (2018). Bivalent Histone Modifications and Development. Curr. Stem Cell Res. Ther..

[97] Liau B.B., Sievers C., Donohue L.K., Gillespie S.M., Flavahan W.A., Miller T.E., Venteicher A.S., Hebert C.H., Carey C.D., Rodig S.J. (2017). Adaptive Chromatin Remodeling Drives Glioblastoma Stem Cell Plasticity and Drug Tolerance. Cell Stem Cell.

[98] Almassalha L.M., Bauer G.M., Wu W., Cherkezyan L., Zhang D., Kendra A., Gladstein S., Chandler J.E., VanDerway D., Seagle B.L. (2017). Macrogenomic Engineering via Modulation of the Scaling of Chromatin Packing Density. Nat. Biomed. Eng..

[99] Sato K., Imai T., Okayasu R., Shimokawa T. (2014). Heterochromatin Domain Number Correlates with X-Ray and Carbon-Ion Radiation Resistance in Cancer Cells. Radiat. Res..

[100] Frame F.M., Pellacani D., Collins A.T., Simms M.S., Mann V.M., Jones D.D., Meuth M., Bristow R.G., Maitland N.J. (2013). HDAC Inhibitor Confers Radiosensitivity to Prostate Stem-Like Cells. Br. J. Cancer.

[101] Lafon-Hughes L., Di Tomaso M.V., Liddle P., Toledo A., Reyes-Ábalos A., Folle G.A. (2013). Preferential Localization of γH2AX Foci in Euchromatin of Retina Rod Cells After DNA Damage Induction. Chromosome Res..

[102] Frame F.M., Noble A.R., Klein S., Walker H.F. (2017). Tumor Heterogeneity and Therapy Resistance-Implications for Future Treatments of Prostate Cancer. J. Cancer Metastasis.

[103] Horsman M.R., Overgaard J. (2016). The Impact of Hypoxia and Its Modification of the Outcome of Radiotherapy. J. Radiat..

[104] Johnson A.B., Barton M.C. (2007). Hypoxia-Induced and Stress-Specific Changes in Chromatin Structure and Function. Mutat. Res. Fund. Mol. Mech. Mutagenesis.

[105] Prickaerts P., Adriaens M.E., van den Beucken T., Koch E., Dubois L., Dahlmans V.E.H., Gits C., Evelo C.T.A., Chan-Seng-Yue M., Wouters B.G. (2016). Hypoxia Increases Genome-Wide Bivalent Epigenetic Marking by Specific Gain of H3K27me3. Epigenet. Chromatin.

[106] Galanis E., Anderson S.K., Miller C.R., Sarkaria J.N., Jaeckle K., Buckner J.C., Ligon K.L., Ballman K.V., Moore D.F., Nebozhyn M. (2018). Phase I/II Trial of Vorinostat Combined with Temozolomide and Radiation Therapy for Newly Diagnosed Glioblastoma: Results of Alliance N0874/ABTC 02. Neuro-Oncology.

[107] Mueller A.C., Sun D., Dutta A. (2013). The miR-99 Family Regulates the DNA Damage Response Through Its Target SNF2H. Oncogene.

[108] Rane J.K., Erb H.H.H., Nappo G., Mann V.M., Simms S., Collins A.T., Visakorpi T., Maitland N.J. (2016). Inhibition of the Glucocorticoid Receptor Results in an Enhanced miR-99a/100-Mediated Radiation Response in Stem-Like Cells from Human Prostate Cancers. Oncotarget.

[109] Mulder K.W., Wang X., Escriu C., Ito Y., Schwarz R.F., Gillis J., Sirokmány G., Donati G., Uribe-Lewis S., Pavlidis P. (2012). Diverse Epigenetic Strategies Interact to Control Epidermal Differentiation. Nat. Cell Biol..

[110] Mateo J., Carreira S., Sandhu S., Miranda S., Mossop H., Perez-Lopez R., Rodrigues D.N., Robinson D., Omlin A., Tunariu N. (2015). DNA-Repair Defects and Olaparib in Metastatic Prostate Cancer. N. Engl. J. Med..

[111] Henneman L., Van Miltenburg M.H., Michalak M., Braumuller T.M., Jaspers J.E., Drenth A.P., de Korte-Grimmerink R., Gogola E., Szuhai K., Schlicker A. (2015). Selective Resistance to the PARP Inhibitor Olaparib in a Mouse Model for BRCA1-Deficient Metaplastic Breast Cancer. Proc. Natl. Acad. Sci. USA.

[112] Thiery J.P., Acloque H., Huang R.Y.J., Nieto M. (2009). Epithelial-Mesenchymal Transitions in Development and Disease. Cell.

[113] Lin K.-T., Yeh Y.-M., Chuang C.-M., Yang S.Y., Chang J.-W., Sun S.-P., Wang Y.-S., Chao K.-C., Wang L.H. (2015). Glucocorticoids Mediate Induction of microRNA-708 to Suppress Ovarian Cancer Metastasis Through Targeting Rap1B. Nat. Commun..

[114] Taplin M.-E., Manola J., Oh W.K., Kantoff P.W., Bubley G.J., Smith M., Barb D., Mantzoros C., Gelmann E.P., Balk S.P. (2008). A Phase II Study of Mifepristone (RU-486) in Castration-Resistant Prostate Cancer, with a Correlative Assessment of Androgen-Related Hormones. BJU Int..

[115] Abeshouse A., Jaeil A., Rehan A., Adrian A., Samirkumar A., Andry C.D., Annala M., Aprikian A., Armenia J., Arora A. (2015). The Molecular Taxonomy of Primary Prostate Cancer. Cell.

[116] Laurent L., Wong E., Li G., Huynh T., Tsirigos A., Ong C.T., Low H.M., Kin Sung K.W., Rigoutsis I., Loring J. (2010). Dynamic Changes in the Human Methylome During Differentiation. Genome Res..

[117] Hawkins R.D., Hon G.C., Lee L.K., Ngo Q.M., Lister R., Pelizzola M., Edsall L.E., Kuan S., Luu Y., Klugman S. (2010). Distinct Epigenomic Landscapes of Pluripotent and Lineage-Committed Human Cells. Stem Cell.

[118] Lee W.H., Morton R.A., Epstein J.I., Brooks J.D., Campbell P.A., Bova G.S., Hsieh W.S., Isaacs W.B., Nelson W.G. (1994). Cytidine Methylation of Regulatory Sequences Near the Pi-Class Glutathione S Transferase Gene Accompanies Human Prostatic Carcinogenesis. Proc. Natl. Acad. Sci. USA.

[119] Nakayama M., Bennett C.J., Hicks J.L., Epstein J.I., Platz E.A., Nelson W.G., De Marzo A.M. (2003). Hypermethylation of the Human Glutathione S-Transferase-Pi Gene (GSTP1) CpG Island Is Present in a Subset of Proliferative Inflammatory Atrophy Lesions but Not in Normal or Hyperplastic Epithelium of the Prostate: A Detailed Study Using Laser-Capture Microdissection. Am. J. Pathol..

[120] Luo J.-H., Ding Y., Chen R., Michalopoulos G., Nelson J., Tseng G., Yu Y.P. (2013). Genome Genome-wide Methylation Analysis of Prostate Tissues Reveals Global Methylation Patterns of Prostate Cancer. Am. J. Pathol..

[121] Aryee M.J., Liu W., Engelmann J.C., Nuhn P., Gurel M., Haffner M.C., Esopi D., Irizarry R.A., Getzenberg R.H., Nelson W.G. (2013). DNA Methylation Alterations Exhibit Intraindividual Stability and Interindividual Heterogeneity in Prostate Cancer Metastases. Sci. Transl. Med..

[122] Angermueller C., Clark S.J., Lee H.J., Macaulay I.C., Teng M.J., Hu T.X., Krueger F., Smallwood S., Ponting C.P., Voet T. (2016). Parallel Single-Cell Sequencing Links Transcriptional and Epigenetic Heterogeneity. Nat. Methods.

[123] Pellacani D., Kestoras D., Droop A.P., Frame F.M., Berry P.A., Lawrence M.G., Stower M.J., Simms M.S., Mann V.M., Collins A.T. (2014). DNA Hypermethylation in Prostate Cancer Is a Consequence of Aberrant Epithelial Differentiation and Hyperproliferation. Cell Death Diff..

[124] Wang Q., Williamson M., Bott S., Brookman-Amissah N., Freeman A., Nariculam J., Hubank M.J.F., Ahmed A., Masters J.R. (2007). Hypomethylation of WNT5A, CRIP1 and S100P in Prostate Cancer. Oncogene.

[125] Yamamoto H., Oue N., Sato A., Hasegawa Y., Matsubara A., Yasui W., Kikuchi A. (2010). Wnt5a Signaling Is Involved in the Aggressiveness of Prostate Cancer and Expression of Metalloproteinase. Oncogene.

[126] Miyamoto D.T., Zheng Y., Wittner B.S., Lee R.J., Zhu H., Broderick K.T., Desai R.I., Fox D.B., Brannigan B.W., Trautwein J. (2015). RNA-Seq of Single Prostate CTCs Implicates Noncanonical Wnt Signaling in Antiandrogen Resistance. Science.

[127] Yu Y.P., Ding Y., Chen R., Liao S.G., Ren B.-G., Michalopoulos A., Michalopoulos G., Nelson J., Tseng G.C., Luo J.-H. (2013). Whole-Genome Methylation Sequencing Reveals Distinct Impact of Differential Methylations on Gene Transcription in Prostate Cancer. Am. J. Pathol..

[128] Pellacani D., Droop A.P., Frame F., Simms M.S., Mann V.M., Collins A.T., Eaves C.J., Maitland N.J. (2018). Phenotype-Independent DNA Methylation Changes in Prostate Cancer. Br. J. Cancer.

[129] Kim J.H., Dhanasekaran S.M., Prensner J.R., Cao X., Robinson D., Kalyana-Sundaram S., Huang C., Shankar S., Jing X., Iyer M. (2011). Deep Sequencing Reveals Distinct Patterns of DNA Methylation in Prostate Cancer. Genome Res..

[130] Doi A., Park I.-H., Wen B., Murakami P., Aryee M.J., Irizarry R., Herb B., Ladd-Acosta C., Rho J., Loewer S. (2009). Differential Methylation of Tissue and Cancerspecific CpG Island Shores Distinguishes Human Induced Pluripotent Stem Cells, Embryonic Stem Cells and Fibroblasts. Nat. Genet..

[131] Heintzman N.D., Hon G.C., Hawkins R.D., Kheradpour P., Stark A., Harp L.F., Ye Z., Lee L.K., Stuart R.K., Ching C.W. (2009). Histone Modifications at Human Enhancers Reflect Global Cell-Type-Specific Gene Expression. Nature.

[132] Thurman R.E., Rynes E., Humbert R., Vierstra J., Maurano M.T., Haugen E., Sheffield N.C., Stergachis A.B., Wang H., Vernot B. (2012). The Accessible Chromatin Landscape of the Human Genome. Nature.

[133] Heinz S., Romanoski C.E.B., Glass C. (2015). The Selection and Function of Cell Type Specific Enhancers. Nat. Rev. Mol. Cell Biol..

[134] Foster C.S., Falconer A., Dodson A.R., Norman A.R., Dennis N., Fletcher A., Southgate C., Dowe A., Dearnaley D., Jhavar S. (2004). Transcription Factor E2F3 Overexpressed in Prostate Cancer Independently Predicts Clinical Outcome. Oncogene.

[135] Li K., Liu C., Zhou B., Bi L., Huang H., Lin T., Xu K. (2013). Role of EZH2 in the Growth of Prostate Cancer Stem Cells Isolated From LNCaP Cells. Int. J. Mol. Sci..

[136] Lyon M.F. (1961). Gene Action in the X-Chromosome of the Mouse (Mus Musculus L.). Nature.

[137] Cheng M.K., Disteche C.M. (2004). Silence of the Fathers: Early X Inactivation. BioEssays.

[138] Hanahan D., Weinberg R.A. (2011). Hallmarks of Cancer: The Next Generation. Cell.

[139] Blackwood J.K., Williamson S.C., Greaves L.C., Wilson L., Rigas A.C., Sandher R., Pickard R.S., Robson C.N., Turnbull D.M., Taylor R.W. (2011). In Situ Lineage Tracking of Human Prostatic Epithelial Stem Cell Fate Reveals a Common Clonal Origin for Basal and Luminal Cells. J. Pathol..

[140] Eckersley-Maslin M.A., Thybert D., Bergmann J.H., Marioni J.C., Flicek P., Spector D.L. (2014). Random Monoallelic Gene Expression Increases Upon Embryonic Stem Cell Differentiation. Dev. Cell.

[141] Deng Q., Ramsköld D., Reinius B., Sandberg R. (2014). Single-Cell RNA-Seq Reveals Dynamic, Random Monoallelic Gene Expression in Mammalian Cells. Science.

[142] Dar R.D., Razooky B.S., Singh A., Trimeloni T.V., McCollum J.M., Cox C.D., Simpson M.L., Weinberger L.S. (2012). Transcriptional burst frequency and burst size are equally modulated across the human genome. Proc. Natl. Acad. Sci. USA.

[143] Patel A.P., Tirosh I., Trombetta J., Shalek A., Gillespie S.M., Wakimoto H., Cahill D.P., Nahed B.V., Curry W.T., Martuza R.L. (2014). Single-Cell RNA-Seq Highlights Intratumoral Heterogeneity in Primary Glioblastoma. Science.

[144] Gendrel A., Attia M., Chen C.-J., Diabangouaya P., Servant N., Barillot E., Heard E. (2014). Developmental Dynamics and Disease Potential of Random Monoallelic Gene Expression. Dev. Cell.

[145] Gimelbrant A., Hutchinson J.N., Thompson B.R., Chess A. (2007). Widespread Monoallelic Expression on Human Autosomes. Science.

[146] Nag A., Vigneau S., Savova V., Zwemer L.M. (2015). Chromatin Signature Identifies Monoallelic Gene Expression Across Mammalian Cell Types. G3.

[147] Dunham I., Kundaje A., Aldred S.F., Collins P.J., Davis C.A., Doyle F., Epstein C.B., Frietze S., Harrow J., Kaul R. (2012). An integrated encyclopedia of DNA elements in the human genome. Nature.

[148] Wang J., Valo Z., Bowers C.W., Smith D.D., Liu Z., Singer-Sam J. (2010). Dual DNA Methylation Patterns in the CNS Reveal Developmentally Poised Chromatin and Monoallelic Expression of Critical Genes. PLoS ONE.

[149] Lin B., Lee H., Yoon J.G., Madan A., Wayner E., Tonning S., Hothi P., Schroeder B., Ulasoc I., Foltz G. (2015). Global Analysis of H3K4me3 and H3K27me3 Profiles in Glioblastoma Stem Cells and Identification of SLC17A7 as a Bivalent Tumor Suppressor Gene. Oncotarget.

[150] Trotman L.C., Niki M., Dotan Z.A., Koutcher J.A., Di Cristofano A., Xiao A., Khoo A.S., Roy-Burman P., Greenberg N.M., van Dyke T. (2003). Pten Dose Dictates Cancer Progression in the Prostate. PLoS Biol..

[151] Wang S., Garcia A.J., Wu M., Aawson D., Witte O.N., Wu H. (2006). Pten Deletion Leads to the Expansion of a Prostatic Stem/Progenitor Cell Subpopulation and Tumor Initiation. Proc. Natl. Acad. Sci. USA.

[152] Whang Y.E., Wu X., Suzuki H., Reiter R.E., Tran C., Vessella R.L., Said J.W., Isaacs W.B., Sawyers C.L. (1998). Inactivation of the Tumor Suppressor PTEN/MMAC1 in Advanced Human Prostate Cancer Through Loss of Expression. Proc. Natl. Acad. Sci. USA.

[153] Suzuki A., de la Pompa J.L., Stambolic V., Elia A.J., Sasaki T., del Barco Barrantes I., Ho A., Wakeham A., Itie A., Fukumoto M. (1998). High Cancer Susceptibility and Embryonic Lethality Associated with Mutation of the PTEN Tumor Suppressor Gene in Mic. Curr. Biol..

[154] Suzuki H., Freije D., Nusskern D.R., Okami K., Cairns P., Sidransky D., Isaacs W.B., Bova B.S. (1998). Interfocal Heterogeneity of PTEN/MMAC1 Gene Alterations in Multiple Metastatic Prostate Cancer Tissues. Cancer Res..

[155] Raval A., Tanner S.M., Byrd C., Angerman E.B., Perko J.D., Chen S.-S., Hackanson B., Grever M.R., Lucas D.M., Matkovic J.J. (2007). Downregulation of Death-Associated Protein Kinase 1 (DAPK1) in Chronic Lymphocytic Leukemia. Cell.

[156] Park D., Zhong Y., Lu Y., Rycaj K., Gong S., Chen X., Liu X., Chao H.P., Whitney P., Calhoun-Davis T. (2016). Stem Cell and Neurogenic Gene-Expression Profiles Link Prostate Basal Cells to Aggressive Prostate Cancer. Nat. Commun..

[157] Wood L.D., Parsons D.W., Jones S., Lin J., Sjöblom T., Leary R.J., Shen D., Boca S.M., Barber T., Ptak J. (2007). The Genomic Landscapes of Human Breast and Colorectal Cancers. Science.

[158] Lawrence M.S., Stojanov P., Polak P., Kryukov G.V., Cibulskis K., Sivachenko A., Carter S.L., Stewart C., Mermel C.H., Roberts S.A. (2013). Mutational Heterogeneity in Cancer and the Search for New Cancer-Associated Genes. Nature.

[159] Rashid N.U., Sperling S., Bolli N., Wedge D.C., Van Loo P., Tai Y.T., Shammas M.A., Fulciniti M., Samur M.K., Richardson P.G. (2014). Differential and Limited Expression of Mutant Alleles in Multiple Myeloma. Blood.

[160] Efeyan A., Serrano M. (2007). P53: Guardian of the Genome and Policeman of the Oncogenes. Cell Cycle.

[161] Finn O.J. (2018). A Believer’s Overview of Cancer Immunosurveillance and Immunotherapy. J. Immunol..

[162] Cerveira N., Ribeiro F.R., Peixoto A., Costa V., Henrique R., Jerónimo C., Teixeira M.R. (2006). TMPRSS2-ERG Gene Fusion Causing ERG Overexpression Precedes Chromosome Copy Number Changes in Prostate Carcinomas and Paired HGPIN Lesions. Neoplasia.

[163] Taoudi S., Bee T., Hilton A., Knezevic K., Scott J., Willson T.A., Collin C., Thomas T., Voss A.K., Kile B.T. (2011). ERG Dependence Distinguishes Developmental Control of Hematopoietic Stem Cell Maintenance from Hematopoietic Specification. Genes Dev..

[164] Cooper C.S., Eeles R., Wedge D.C., Van Loo P., Gundem G., Alexandrov L.B., Kremeyer B., Butler A., Lynch A.G., Camacho N. (2015). Analysis of the Genetic Phylogeny of Multifocal Prostate Cancer Identifies Multiple Independent Clonal Expansions in Neoplastic and Morphologically Normal Prostate Tissue. Nat. Genet..

